# The Influence of *Nasturtium officinale* R. Br. Agar and Agitated Microshoot Culture Media on Glucosinolate and Phenolic Acid Production, and Antioxidant Activity

**DOI:** 10.3390/biom10091216

**Published:** 2020-08-21

**Authors:** Marta Klimek-Szczykutowicz, Agnieszka Szopa, Michał Dziurka, Łukasz Komsta, Michał Tomczyk, Halina Ekiert

**Affiliations:** 1 Chair and Department of Pharmaceutical Botany, Faculty of Pharmacy, Medical College, Jagiellonian University, ul. Medyczna 9, 30-688 Kraków, Poland; marta.klimek-szczykutowicz@doctoral.uj.edu.pl (M.K.-S.); halina.ekiert@uj.edu.pl (H.E.); 2 Polish Academy of Sciences, The Franciszek Górski Institute of Plant Physiology, ul. Niezapominajek 21, 30-239 Kraków, Poland; m.dziurka@ifr-pan.edu.pl; 3 Department of Medicinal Chemistry, Faculty of Pharmacy with Division of Medical Analytics, Medical University of Lublin, ul. Chodźki 4a, 20-093 Lublin, Poland; lukasz.komsta@umlub.pl; 4 Department of Pharmacognosy, Faculty of Pharmacy with the Division of Laboratory Medicine, Medical University of Białystok, ul. Mickiewicza 2a, 15-230 Białystok, Poland; michal.tomczyk@umb.edu.pl

**Keywords:** in vitro cultures, plant growth regulators, phenolic acids, glucosinolates, antioxidant potential

## Abstract

This paper presents an optimization of conditions for microshoot cultures of *Nasturtium officinale* R. Br. (watercress). Variants of the Murashige and Skoog (MS) medium containing different plant growth regulators (PGRs): cytokinins—BA (6-benzyladenine), 2iP (6-γ,γ-dimethylallylaminopurine), KIN (kinetin), Zea (zeatin), and auxins—IAA (3-indoleacetic acid), IBA (indole-3-butyric acid), 2,4-d (2,4-dichlorophenoxyacetic acid), IPA (indole-3-pyruvic acid), NAA (naphthalene-1-acetic acid), total 27 MS variants, were tested in agar and agitated cultures. Growth cycles were tested for 10, 20, or 30 days in the agar cultures, and 10 or 20 days in the agitated cultures. Glucosinolate and phenolic acid production, total phenolic content and antioxidant potential were evaluated. The total amounts of glucosinolates ranged from 100.23 to 194.77 mg/100 g dry weight of biomass (DW) in agar cultures, and from 78.09 to 182.80 mg/100 g DW in agitated cultures. The total phenolic acid content varied from 15.89 to 237.52 mg/100 g DW for the agar cultures, and from 70.80 to 236.74 mg/100 g DW for the agitated cultures. Extracts of the cultured biomass contained higher total amounts of phenolic acids, lower total amounts of glucosinolates, a higher total phenolic content and similar antioxidant potentials compared to plant material. The analyses performed confirmed for the first time the explicit influence on secondary metabolite production and on the antioxidant potential. The significance was statistically estimated in a complex manner.

## 1. Introduction

*Nasturtium officinale* R. Br. (Robert Brown, described earlier by Linnaeus as *Sisymbrium nasturtium-aquaticum*), called watercress, belongs to the family *Brassicaceae*. It is a rare perennial, aquatic or semi-aquatic herb with characteristic creeping or floating stems. In its natural habitat it colonizes gently flowing and shallow streams. *N. officinale* is native to Europe, North Africa and Asia [1]. In Europe, this species has been classified by the International Union for Conservation of Nature (IUCN) Red List of Threatened Species, as a plant of least concern. However, the classification of *N. officinale* is different in each European country; for example, it is considered an extinct or probably extinct plant in Estonia, endangered in Austria and Sweden, and partly endangered in Poland [2]. The monograph of the *N. officinale* herb is present in German Commission E Monographs (Phyto-Therapy) [3], and it also has an important position in German Commission D Monographs [4] for homeopathic medicines. Additionally, the European Food Safety Authority (EFSA) has classified this species as a safe vegetable in the group of “leaf vegetables, herbs and edible flowers” [5]. Its medicinal effects were used in the traditional medicine of Iran, Azerbaijan, Morocco and Mauritius to treat hyperglycemia, hypertension, asthma and cough [6,7,8]. Current studies have confirmed a wide spectrum of biological activity determined by a rich chemical composition, such as anticancer, antioxidant, antimicrobial, anti-inflammatory, antipsoriatic and cardioprotective [9,10,11,12]. The main groups of compounds found in the herb are: glucosinolates, isothiocyanates, polyphenols (flavonoids, phenolic acids, proanthocyanidins), terpenoids (including carotenoids), vitamins (B1, B2, B3, B6, E, C) and bioelements [13,14,15,16,17]. *N. officinale* is also used in the cosmetic industry as an antioxidant, anti-ageing, skin-lightening and anti-acne agent [18]. *N. officinale* is also increasingly popular in modern cuisine. Currently, it is added to European dishes such as salads and soups because of its rich composition and numerous scientifically proven properties [1]. Moreover, it has aproven valuable phytoremediation capacity. Current studies have confirmed its ability to purify water and remove copper (Cu), nickel (Ni), zinc (Zn) [19], cadmium (Cd), cobalt (Co), chromium (Cr) [20,21], arsenic (As) [22] and uranium (U) [23] from soil.

Plant biotechnology facilitates controlled in vitro plant cultures with conditions optimized to cultivate and propagate *N. officinale* (micropropagation). The optimization process concerns factors such as components of the growth medium, plant growth regulators (PGRs), and culture type (agar, agitated, bioreactor) for a given plant species. Its purpose is to increase, as much as possible, the accumulation of secondary metabolites in the biomass of in vitro cultures. In plant biotechnology, to increase the amounts of these compounds, selection of the medium, elicitors, and also genetic transformations of high-productivity cell lines can be used. In vitro plant cultures may be an alternative source of secondary metabolites to plants grown in the traditional way. This creates possibilities for endangered plants, whose numbers are rapidly decreasing [24].

*N. officinale* in vitro cultures are the object of a few research papers, which mainly focus on micropropagation and the plant’s ability to regenerate from callus cultures [25]. The literature on biotechnological studies of *N. officinale* also includes the influence of genetic transformation by *Rhizobium rhizogenes* on the production of glucosinolates. Recent research conducted by our team also proved the ability of in vitro cultured microshoots to accumulate several bioelements such as calcium, chromium, copper, iron, lithium, magnesium, selenium and zinc, and the influence of the culture medium on the production of glucosinolates and phenolic acids, as well as on the antioxidant activity of biomass extracts [26]. The aim of the present research was to investigate the influence of the qualitative and quantitative composition of exogenous auxins and cytokinins in the culture medium on biomass growth and production of various important metabolites.

## 2. Materials and Methods

### 2.1. Parent Plant Material

For comparative purposes, analyses of the parent plant were performed. As a plant material, the *N. officinale* R. Br. herb harvested from the Garden of Medicinal Plants, Faculty of Pharmacy, Medical College, Jagiellonian University, Kraków, Poland, in May 2017 was used. After collecting the material, it was lyophilized (Labconco lyophilizer, Kansas City, KS, USA) and analyzed.

### 2.2. Initiation of In Vitro Cultures

*N. officinale* microshoot cultures were initiated and maintained as reported previously [26]. The initiating medium was standard MS medium [27] with 0.72% (*w*/*v*) agar (plant agar, Duchefa, Haarlem, Netherlands), 3% (*w*/*v*) sucrose, containing 1 mg/L each cytokinin and auxin, respectively BA, and NAA. After approximately two weeks, viable, green, single microshoot cultures were obtained, which were transferred to a new medium. *N. officinale* microshoot cultures were kept in the following conditions: 25 ± 2 °C continuous illumination by LED white light with a photosynthetic photon flux density (PPFD) of 40 µmol m^−2^ s^−1^, and they were passaged at 2-week intervals.

### 2.3. Experimental In Vitro Cultures

#### 2.3.1. Agar Microshoot Cultures

**Figure 1:**
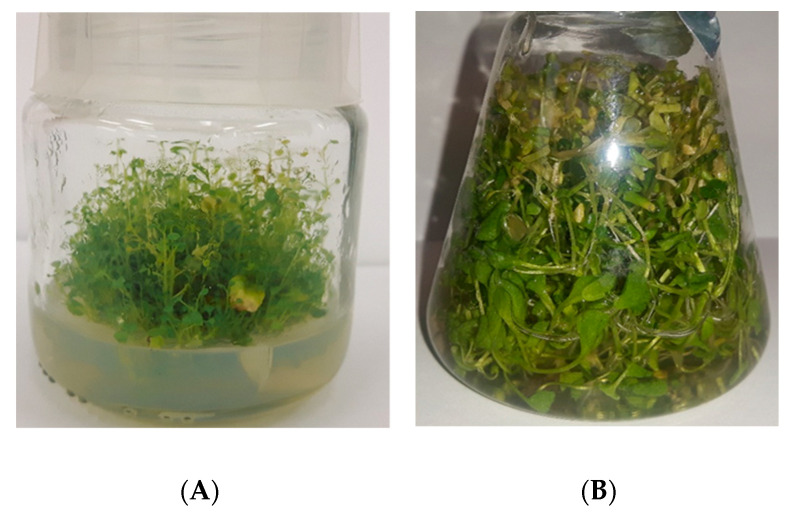
The microshoot cultures of *N. officinale*. (**A**) Agar microshoot culture; MS medium containing 1 mg/L BA and 1 mg/L NAA, 30th day of culture growth. (**B**) Agitated microshoot culture; MS medium containing 1 mg/L Zea and 1 mg/L NAA, 20th day of culture growth.

Stationary agar microshoot cultures (Figure 1A) were kept on several variants of the standard MS [27] medium with 0.72% (*w*/*v*) agar and 3% (*w*/*v*) sucrose. The amount of inoculum was equal to 1 g of microshoots. The experiment was conducted in Magenta™ vessels (Sigma-Aldrich, product No. V8630, Poznań, Poland) with an addition 30 mL of culture medium. The first experiments involved basal studies on the optimization of MS media variants containing one cytokinin as a PGR: BA, 2iP, KIN, or Zea alone, at a concentration of 1 mg/L, and the control—without the addition of PGR (i.e., four MS medium variants and control) (Tables S1, S2 and S6). Subsequently, the good results with the application of BA as cytokinin prompted us to test different combinations of BA as cytokinin and NAA as auxin in the respective concentrations of 2 and 1, 1 and 2, 1 and 1 (mg/L) (i.e., three MS medium variants). The results of this experiment (Tables S2 and S7) proved that the 1:1 ratio of cytokinin to auxin was optimal. According to these results, the agar microshoot cultures were further tested with different combinations of 4 cytokinins: BA, 2iP, KIN and Zea, and 5 auxins: IAA, IBA, IPA, 2,4-d, and NAA, all at a concentration of 1 mg/L each (i.e., 20 MS medium variants) (Table S1). In total, 27 MS medium variants containing different combinations of PGRs were tested. The cultures were grown in 10, 20 and 30-day cycles (3 series, *n* = 5). Microshoots were grown under continuous LED white light, at PPFD of 40 μmol m^−2^ s^−1^, at 25 ± 2 °C.

#### 2.3.2. Agitated Microshoot Cultures 

The experiments with agitated microshoot cultures (Figure 1B) were conducted in 100 mL Erlenmeyer flasks. Each flask contained 50 mL of MS medium. In these studies a rotary shaker (Elpin-Plus, Lubawa, Poland) was used at 150 rpm. For agitated cultures, the inoculum included 0.5 g microshoot sections. The cultures used six variants of the standard liquid MS [27] medium with 3% (*w*/*v*) sucrose, containing respectively: 1 mg/L cytokinin and 1 mg/L auxin—BA and NAA, 2iP and NAA, KIN and IAA, KIN and IBA, Zea and IBA, Zea and NAA. This choice of PGR combinations was made based on the fact that these variants had given the best growth and production results in the agar cultures. The experimental media and biomass samples were collected from the agitated cultures at 10-day intervals on day 10 and day 20 (after 30 days the cultures died and were not tested). Microshoots were grown under continuous LED white light, at PPFD of 40 μmol m^−2^ s^−1^, at 25 ± 2 °C. A total of three series were performed for each experiment (*n* = 5).

### 2.4. Biomass Growth

The initial weight (inoculum) in the experiments was 1 g for agar cultures and 0.5 g for agitated cultures. The durations of the growth period were 10, 20 and 30 days for agar cultures, and 10 and 20 days for agitated cultures. Fresh and dry biomass growth was recorded by weighing the fresh biomass obtained after harvesting and the dry biomass obtained after lyophilization (Labconco lyophilizer, Kansas City, KS, USA), respectively. Dry biomass increments were calculated based on the growth index (Gi) according to the formula: Gi=$\;\frac{\left(Dw_{1}-Dw_{0}\right)}{Dw_{0}}\;$, where Dw_1_ is the dry weight of microshoots at the end of the experiment, and Dw_0_ is the dry weight of the inoculum [28].

### 2.5. Spectrophotometric Analysis of the Total Glucosinolate Pool

The analysis involved several MS medium variants, both for agar and agitated cultures: BA and NAA, 2iP and NAA, KIN and IAA, Zea and IBA, Zea and NAA. Each of the agar cultures was tested after a growth period of 10, 20 and 30 days, and agitated cultures after 10 and 20 days. A control without PGRs was also tested in agar after 10, 20 and 30 days of culture. Glucosinolate analysis was performed with the method developed by Gallaher et al. [29]. Lyophilized samples were pulverized (MM400, Retch, Arzberg, Germany) and two sets of 15 mg samples were prepared. The first set of samples was extracted under inactivated myrosinase conditions. The samples were heated for 10 min. at 80 °C. Subsequently, 0.85 mL of methanol was added and the samples were further heated for 20 min. After that, 0.2 mL of boiling H_2_O was added and heating was continued for 30 min. The samples were then shaken (5 min, 30 Hz, MM400), centrifuged (5 min., 22,000× *g*, Universal32R, Hettich, Kirchlengern, Germany) and the supernatant was collected. Double extraction with an additional 1 mL of 90% methanol was carried out. During the analysis, extraction was also performed for a second set of samples under active myrosinase conditions. Water (0.2 mL) was added to the samples. Then 0.85 mL methanol after 30 min. in ambient conditions was used. The extraction of samples was carried out at ambient temperature as described above. Glucosinolate stability at 80 °C was measured using sinigrin recovery. The supernatants were combined and N_2_ at 45 °C (TurboVap, Zymark, Midland, MI, USA) was used to dry the extracts. The storage temperature of the extracts was −20 °C until further processing. SPE anion exchange columns (Supel-Select SAX, 60 mg, 3 mL, Supelco, Midland, MI, USA) were used to purify glucosinolates. First, the columns were activated by adding 0.5 mL of methanol, followed by the addition of 0.5 mL of water, 0.5 mL of 0.5 M sodium acetate (pH 4.6), and 0.5 mL of water. Then, glucosinolates were eluted with 4 × 0.25 mL of 0.5 M NaCl. The eluate was dried under N_2_, and glucosinolates were hydrolyzed by adding 0.5 mL of freshly prepared 1 M sodium hydroxide. Then, the neutralization reaction in the samples was carried out using 40 μL of concentrated hydrochloric acid after 30 min. The colorimetric reaction was initiated by adding 125 μL of 2 mM potassium ferricyanide solution in 0.4 M phosphate buffer (pH 7.0) to 125 μL of the standard solution or sample extract in 96-well plate format. Absorbance was read at 420 nm 2 min. after adding ferricyanide solution (Synergy II, BioTek, Winooski, VT, USA). Sinigrin was used as a calibration standard. The results of the total glucosinolate content are expressed as mg of sinigrin/100 g dry weight (DW).

### 2.6. Phenolic acid Analysis

The analysis involved variants of MS medium for the agar cultures containing: 1 mg/L BA, 1 mg/L 2iP, 1 mg/L KIN, 1 mg/L Zea, control (without PGRs), 2 mg/L BA and 1 mg/L NAA, 1 mg/L BA and 2 mg/L NAA, 1 mg/L BA and 1 mg/L NAA; and also different combinations of four cytokinins: BA, 2iP, KIN and Zea, and 5 auxins: IAA, IBA, IPA, 2,4-D and NAA, all at a concentration of 1 mg/L each (i.e., 20 MS medium variants) (Table S1), after a growth period of 10, 20 and 30 days. The tested media for the agitated cultures included MS medium variants containing respectively: 1 mg/L cytokinin and 1 mg/L auxin—BA and NAA, 2iP and NAA, KIN and IAA, KIN and IBA, Zea and IBA, Zea and NAA, after 10 and 20 days. For the analysis, methanolic extracts were used, which were prepared with 0.2 g of lyophilized biomass and weighed-out pulverized biomass from in vitro cultures. Methanol (4 mL) (STANLAB, Lublin, Poland) was added to each sample and ultrasonic bath (POLSONIC 2, Poznań, Poland) was used for extraction twice for 20 min. Before HPLC analysis, sterilizing syringe filters (0.22 μm, Millex^®^GP, Millipore) were used to purify the extracts. The analysis of phenolic acids was performed with the HPLC-DAD method [30,31], using the Merck-Hitachi System and a Purospher RP-18e analytical column (4 × 250 nm, 5 mL; Merck, Darmstadt, DE-HE, Germany). The mobile phase consisted of: A—methanol: 0.5% acetic acid (1:4 *v*/*v*); B—methanol, with the following gradient: 0–20 min., 0% B, 20–35 min., 0–20% B, 35–45 min., 20–30% B, 45–55 min., 30–40% B, 55–60 min., 40–50% B, 60–65 min., 50–75% B and 65–70 min., 75–100% B, with a hold time of 15 min., at 25 °C. The flow rate was 1 mL/min, injection volume was 10 μL. The measurement was taken at a wavelength of 254 nm. Qualitative and quantitative analyses were carried out by comparing the standards of the following phenolic acids (27 compounds): caffeic, caftaric, chlorogenic, *m*-coumaric, *o*-coumaric, p-coumaric, cryptochlorogenic, 3,4-dihydroxyphenylacetic, ellagic, ferulic, gallic, gentisic, hydrocaffeic, *p*-hydroxybenzoic, isochlorogenic, isoferulic, neochlorogenic, phenylacetic, 3-phenylacetic, protocatechuic, rosmarinic, salicylic, sinapic, syringic and vanillic acids, as well as the precursors: benzoic and cinnamic acids (all compounds purchased from Sigma-Aldrich, Saint Louis, MO, USA).

### 2.7. Total Phenolic Assay

The analyses involved variants of MS medium for the agar cultures containing: control (without PGRs), 2 mg/L BA and 1 mg/L NAA, 1 mg/L BA and 2 mg/L NAA, 1 mg/L BA and 1 mg/L NAA; and 1 mg/L each of cytokinin and auxin: 2iP and NAA, KIN and IAA, KIN and IBA, Zea and IBA, Zea and NAA, after a growth period of 10, 20 and 30 days. The tested media for agitated cultures included MS medium variants containing respectively: 1 mg/L cytokinin and 1 mg/L auxin—BA and NAA, 2iP and NAA, KIN and IAA, KIN and IBA, Zea and IBA, Zea and NAA, after 10 and 20 days of culture. Total phenolic content was measured with the Folin–Ciocalteu (F-C) method according to the approach of Singleton et al. [32], with modifications reported by Bach et al. [33]. An aliquot of the extract (100 μL) was added to 0.45 mL of diluted F-C reagent (5:2; *v*/*v*) and after 10 min., 0.45 mL saturated Na_2_CO_3_ was added. Samples were mixed after 2 h incubation (25 °C) in darkness and centrifuged. Absorbance was read at 760 nm. The total phenolic content was expressed as mmol trolox equivalents (TE)/100 g DW. The measurements from each series were performed in five replicates.

### 2.8. Antioxidant Capacity

The analyses involved variants of MS medium for the agar cultures containing: control (without PGRs), 2 mg/L BA and 1 mg/L NAA, 1 mg/L BA and 2 mg/L NAA, 1 mg/L BA and 1 mg/L NAA; and 1 mg/L each of cytokinin and auxin: 2iP and NAA, KIN and IAA, KIN and IBA, Zea and IBA, Zea and NAA, after 10, 20 and 30 days of culture. The tested media for the agitated cultures included MS medium variants containing respectively: 1 mg/L cytokinin and 1 mg/L auxin—BA and NAA, 2iP and NAA, KIN and IAA, KIN and IBA, Zea and IBA, Zea and NAA, after 10 and 20 days. The plant material was lyophilized and homogenized.

#### 2.8.1. CUPRAC Total Antioxidant Capacity Assay

The CUPric Reducing Antioxidant Capacity (CUPRAC) method [34] modified by Biesaga-Kościelniak et al. [35] was used to obtain antioxidant activity in the tested biomass extracts. Extraction was carried out using 5 mg of accurately weighed pulverized samples and 1 mL of methanol (15 min. shaking at 30 Hz, MM400, Retch, Arzberg, BY, Germany). After centrifugation (5 min. at 22,000× *g*), 50 μL aliquots of the extract were transferred into the wells of a 96-well plate prefilled with the same aliquots of 10 mmol/L Cu^2+^, 7.5 mM neocuproine and 1 mol/L ammonia-acetate buffer (pH 7.0). Then the plates were shaken for 15 min. at 25 °C. Absorbance measurements were λ = 425 nm using a Synergy 2 microplate reader (BioTek, Winooski, VT, USA). The amounts of antioxidants were expressed as mmol TE/100 g DW. The measurements for each series were taken in five replicates.

#### 2.8.2. Ferric Reducing Ability (FRAP) Assay 

Ferric reducing ability of plant extracts was measured based on the ferric reducing ability of plasma (FRAP) method by Benzie and Strain [36]. Analyses were done in a 96-well plate format. A solution of 10 mmol/L TPTZ (2,4,6-tris(2-pyridyl)-s-triazine) in 40 mmol/L HCl was mixed with 20 mmol/L FeCl_3_ × 6H_2_O and 300 mmol/L acetate buffer, pH 3.6 at a ratio of 1:1:10 *v*/*v*. Then 50 µL of plant extract was added to 150 µL of that mixture. Samples were mixed and incubated for 5 min. at 25 °C. Absorbance readings were taken at 593 nm. The total amounts of antioxidants were expressed as mmol TE/100 g DW. The measurements from each series were performed in five replicates.

#### 2.8.3. 1,1-Diphenyl-2-Picrylhydrazyl (DPPH) Radical-Scavenging Activity Assay

Free radical-scavenging activity measurements were done using the stable free radical 1,1-diphenyl-2-picrylhydrazyl (DPPH) [37]. 50 µL of plant extract was added to 150 µL of 100 µM DPPH methanolic solution. Samples were incubated for 60 min. at 25 °C in a horizontal shaker in darkness. After that, absorbance was read at 517 nm. The total amounts of antioxidants were expressed as TE in mmol/100 g DW. The measurements from each series were performed in five replicates.

All chemical used for analyses, if otherwise not specified, were of analytical grade supplied by Sigma-Aldrich (Poznań, Poland).

### 2.9. Statistical Model Fitting

The obtained dataset was analyzed statistically inside the R Computational environment (version 3.6.3, ×64, R Core Team, Vienna, Austria). The built-in “lm” function was used to fit linear models. Optimization was done against Akaike Information Criterion (AIC) with the “stepAIC” function from the built-in MASS package. For each experimental property, the following terms were included in the model: intercept term, time as a quantitative variable (in days), each added compound, as a quantitative variable (in milligrams), as well as two-term interactions between compounds and between each compound and time. Validation of fitted models were done using Kolmogorov–Smirnoff test (normality of the residuals), Breusch–Pagan test (heteroskedascity), as well as Mandel test (curvilinearity). Only several models showed significant deviances from these assumptions, but the visual inspection still found them as useful.

## 3. Results

### 3.1. The Experimental In Vitro Cultures

#### 3.1.1. Microshoot Appearance and Biomass Growth

##### Agar Microshoot Cultures

The first experiments involved basal studies of MS media with the addition of one of the following cytokinins: BA, 2iP, KIN, Zea, at a concentration of 1 mg/L each. The control medium, without any addition of PGRs, was used for comparison. In the experiment shown, small increases of biomass and the number of microshoots in the in vitro cultures growing in the medium with the addition of 1 mg/L of one cytokinin only were observed. Variants containing 1 mg/L Zea and 1 mg/L BA and the control medium were characterized by a higher number of microshoots and the dark green color of the shoots and leaves. The Gi values obtained ranged from 0.05 to 3.26. The maximum values were for microshoots growing for 20 days on variant 0 (control) of the medium. A high Gi (2.55) was also obtained for in vitro cultures growing on the MS medium variant containing 1 mg/L BA, after 30 days (Figure S1, Table S2). Good results were obtained with the use of BA as a cytokinin and NAA as an auxin in different concentrations: 2 and 1, 1 and 2, 1 and 1 (mg/L), respectively. Microshoots growing on all these variants were characterized by a large number of microshoots and the dark green color of the leaves. The highest Gi (5.05) was observed after 30 days on the MS medium variant containing 1 mg/L BA and 1 mg/L NAA (Figure S1, Table 1 and Table S2).

The next stage was a study of the effect of different types and concentrations of PGRs added to the MS basal medium. After 10, 20 and 30 days it was checked for any increase in biomass. During the experiment the influence of the type of PGRs added to the MS medium and the duration of the growth period on the appearance of the fresh microshoot biomass was observed (Figures S2–S4). Microshoots that grew on the media containing 1 mg/L 2,4-D died after 10 days of culture. Those variants were not investigated further. *N. officinale* in vitro cultures growing for 20 and 30 days on the following medium variants were characterized by the largest number of green shoots and biomass growth (1 mg/L each of cytokinin and auxin): Zea and NAA, Zea and IBA, BA and NAA, BA and IAA, 2iP and IAA, 2iP and IBA. The values of Gi ranged from 0.18 to 5.05. The highest values of Gi were obtained for microshoots cultivated for 30 days on the MS medium variant containing 1 mg/L BA and 1 mg/L NAA (Figures S2–S4, Table 1 and Table S2).

To sum up the experiments, a comparison of the lowest and highest Gi after various growth cycles showed that the highest 35-fold increase was obtained on the medium variant containing 1mg/L BA and 1 mg/L NAA after 10 days of culture. The lowest difference in the Gi value, of 3.95, was obtained after 30 days, which means that all of the variants achieved high satisfactory biomass increases. The variant containing 1 mg/L BA and 1 mg/L NAA was chosen as the “best growth” medium variant, with the highest biomass growth increments obtained over each growth cycle. The highest growth increments obtained on the medium variant with 1 mg/L BA and 1 mg/L NAA were 1.5 times higher than in the control (Figures S2–S4, Table 1 and Table S2).

##### Agitated Microshoot Cultures

In the agitated microshoot cultures the influence of the MS medium variant and the duration of the culture cycle on the appearance of the biomass were also observed. (Figure S5). Six variants of the MS medium were tested. The highest number of green microshoots was observed after 20 days of growth on the medium containing 1 mg/L Zea and 1 mg/L NAA. After 30 days of culture, the biomass died in all the MS medium variants. For this reason, they were not further tested (Figure S5). The increase of dry biomass calculated by the Gi ranged from 1.47 to 10.48. The differences depended on the medium variant and culture duration. The highest increases were obtained for cultures cultivated for 20 days on the variant containing 1 mg/L Zea and 1 mg/L NAA. Comparing the lowest and highest values of Gi after the various growth cycles, the highest 6.59-fold increase was obtained after 20 days of culture. The lowest growth of 3.60 times was obtained after 10 days, which means that all of the variants achieved satisfactory biomass increases. The variant containing 1 mg/L Zea and 1 mg/L NAA was chosen as the best medium variant, with the highest biomass growth increments obtained in each growth cycle (Figure S5, Table 1 and Table S3).

##### Statistical Analysis

After preliminary calculations, we decided to combine the agar and agitated datasets and to perform a combined statistical analysis, adding another category variable distinguishing these two kinds of cultures (Tables S4 and S5). In the overall analysis, time was a significant factor and biomass increases over time. In general, the significance of all these factors (even after model reduction) was not so high. The amount of obtained biomass was slightly lower in the agitated cultures, but this technique gave a significantly higher slope (growth per day). Only KIN (and 2iP for agitated cultures) significantly lowered biomass growth when all the results were analyzed in combination. The most significant interaction was between IBA and BA.

#### 3.1.2. Effect of Supplementation with Different PGRs on Glucosinolate Production

##### Agar Microshoot Cultures

**Figure 2:**
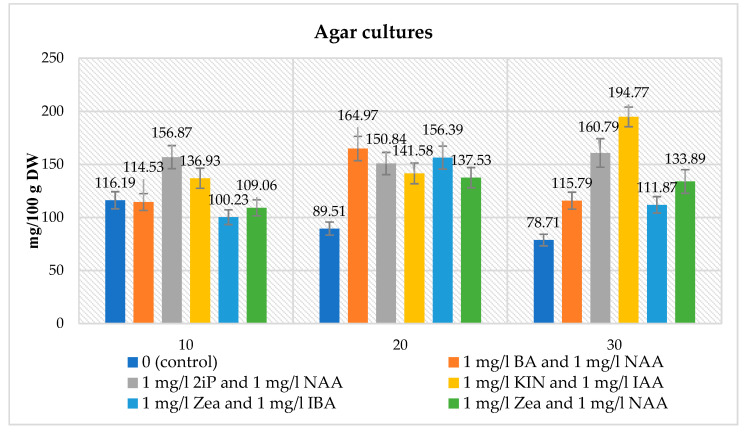
The maximal total glucosinolate contents (mg/100 g DW ± SD) in the extracts from agar *N. officinale* microshoot cultures of tested different MS medium variants and growth cycles.

A spectrophotometric analysis in the biomass extracts of agar microshoot cultures was performed to obtain the quantitative glucosinolate content. It was done for media containing different PGRs at a concentration of 1 mg/L cytokinin and 1 mg/L auxin, respectively for: BA and NAA, 2iP and NAA, KIN and IAA, Zea and IBA, Zea and NAA. For comparative purposes the analysis of the glucosinolate content in the control medium was also performed (without the addition of PGRs). The glucosinolate content varied depending on the medium variant used and the duration of the growth period. The total glucosinolate content ranged from 100.23 to 194.77 mg/100 g DW. For the control sample, the glucosinolate content ranged from 78.71 to 116.19 mg/100 g DW (Figure 2).

The maximal content was obtained for the medium containing 1 mg/L KIN and 1 mg/L IAA after 30 days of culture, whereas the lowest was recorded for the medium variant containing 1 mg/L Zea and 1 mg/L IBA cultured for 10 days. After 10 days, higher amounts of glucosinolate, compared to the control (116.19 mg/100 g DW), were obtained for the medium variants containing, respectively, 1 mg/L each of 2iP and NAA (156.87 mg/100 g DW), KIN and IAA (136.93 mg/100 g DW). Lower glucosinolate amounts were obtained for medium variants containing, respectively, 1 mg/L each of Zea and IBA (100.23 mg/100 g DW), Zea and NAA (109.06 mg/100 g DW), BA and NAA (114.53 mg/100 g DW). After 20 and 30 days, higher total glucosinolate amounts (89.51 mg/100 g DW—20 days, 78.71 mg/100 g DW—30 days) were obtained, compared to the controls, for all the medium variants tested (Figure 2).

##### Agitated Microshoot Cultures

**Figure 3:**
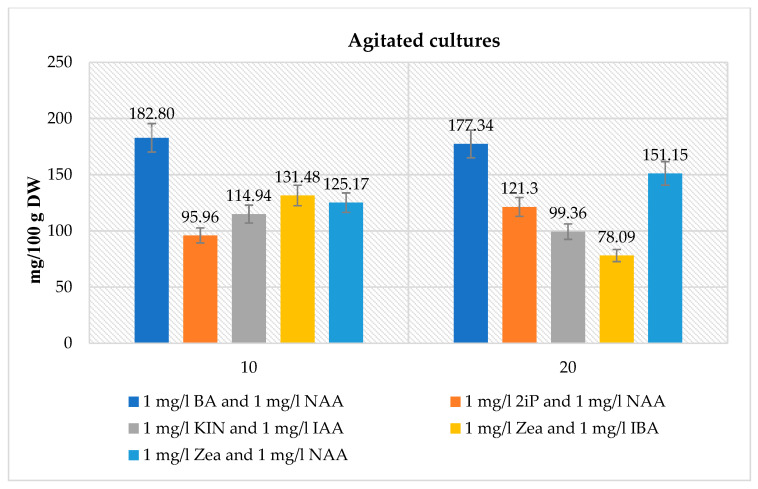
The maximal total glucosinolate contents (mg/100 g DW ± SD) in the extracts from agitated *N. officinale* microshoot cultures of tested different MS medium variants and growth cycles.

In the biomass of tested agitated microshoot cultures cultivated on medium variants containing different PGRs at a concentration of 1 mg/L cytokinin and 1 mg/L auxin, respectively: BA and NAA, 2iP and NAA, KIN and IAA, Zea and IBA, Zea and NAA, quantitative analyses of glucosinolates were performed. The amounts of glucosinolates were dependent on the medium used and the duration of the growth period. The total glucosinolate content ranged from 78.09 to 182.80 mg/100 g DW (Figure 3). The maximal total content was observed for the medium variant containing 1 mg/L BA and 1 mg/NAA after 10 days of culture (182.80 mg/100 g DW). The lowest glucosinolate production was recorded for the medium variant containing 1 mg/L Zea and 1 mg/l IBA after a 20-day growth period –78.09 mg/100 g DW (Figure 3).

##### Statistical Analysis

The statistical analysis was done, as previously, for the combined dataset with the additional category variable representing the type of culture, which was found to be insignificant. The results (Tables S4 and S5) show that the culture type was not incorporated into the final model. BA, Zea, IAA and 2iP slightly (but significantly) lower mean glucosinolate production and increase the slope (production in time per day). No other significant interactions or terms were found in this dataset.

#### 3.1.3. Effect of PGRs on Phenolic Acid Production

##### Agar Microshoot Cultures

Ten phenolic acids (caffeic, *o*-coumaric, *p*-coumaric, ellagic, ferulic, gallic, isoferulic, protocatechuic, rosmarinic and syringic) were identified among the 27 compounds analyzed in the biomass extracts from *N. officinale* agar microshoot cultures grown on MS medium variants containing 1 mg/L cytokinin (four variants), various concentrations of BA and NAA (four variants), and 1 mg/L cytokinin and 1 mg/L auxin (19 variants), using the HPLC-DAD analysis (Figure S6). The significant effect of the supplemented PGRs in the medium variant tested on the accumulation of phenolic acid in the biomass was observed over different growth periods (10, 20, 30 days). The amounts of the individual compounds ranged from 0.02 to 138.40 mg/100 g DW (Table 2 and Table S6–S11). The quantitatively dominant phenolic acids were protocatechuic acid (max. 138.40 mg/100 g DW, 2 mg/L BA and 1 mg/L NAA, 20 days) and gallic acid (max. 61.03 mg/100 g DW, 2 mg/L BA and 1 mg/L NAA, 30 days) (Table 2 and Table S6–S11). The results show that the medium variant and growth period influenced the total amount of the estimated phenolic acids, and ranged from 15.89 (1 mg/L BA, 20 days) to 237.52 mg/100 g DW (2 mg/L BA and 1 mg/L NAA, 20 days) (Table 2 and Table S6–S11). In the control sample, the amounts of individual compounds ranged from 0.13 to 20.49 mg/100 g DW. The main compounds were gallic acid (20.49 mg/100 g DW, 30 days) and protocatechuic acid (10.84 mg/100 g DW, 30 days). The total amount of phenolic acids obtained in the control sample ranged from 48.13 to 55.76 mg/100 g DW (Table S6).

##### Agitated Microshoot Cultures

A total of 10 phenolic acids (caffeic, *o*-coumaric, *p*-coumaric, ellagic, ferulic, gallic, isoferulic, protocatechuic, rosmarinic and syringic) were identified among the 27 compounds analyzed in the biomass extracts from *N. officinale* agitated microshoot cultures grown on MS media supplemented with 1 mg/L cytokinin and 1 mg/L auxin (six variants), using the HPLC-DAD analysis (Figure S6). The presence of the same compounds as those in the agar cultures was confirmed. The HPLC-DAD analyses show a significant influence of the combination PGRs used on the amounts of the individual compounds as well as on their total amounts. The effect of the tested PGRs on the production of phenolic acids in the microshoot biomass was noticeable and also depended on the duration of the growth period (10 and 20 days). The amounts of individual compounds ranged from 0.02 to 132.26 mg/100 g DW (Table 3 and Table S12). Quantitatively dominant phenolic acids were: protocatechuic acid (max. 132.26 mg/100 g DW, 1 mg/L Zea and 1 mg/L NAA, 10 days) and gallic acid (max. 53.34 mg/100 g DW, 1 mg/L Zea and 1 mg/L NAA, 10 days) (Table 3 and Table S12). The total amount of the detected phenolic acids was dependent on the medium variant and growth period, and ranged from 70.80 (1 mg/L KIN and 1 mg/L IAA, 10 days) to 236.74 mg/100 g DW (1 mg/L Zea and 1 mg/L NAA, 10 days) (Table 3 and Table S12).

##### Statistical Analysis

Analyzing the whole dataset statistically (Tables S4 and S5) one can conclude that:

1. The amounts of gallic, protocatechuic, caffeic, and syringic acids, and the total amount of phenolic acids were significantly higher in the agitated cultures under the same conditions.
2. Additions of BA, NAA, Zea, IAA, 2iP, KIN, 2,4-D and IBA caused an increase in many compounds and sometimes a decrease in *p*-coumaric, *o*-coumaric, or ferulic acids. This change was correlated with an opposite change of the slope against time (when average content is higher, production in time is lower, and *vice versa*). For the agitated cultures, the addition of NAA did not much increase the synthesis of gallic, protocatechuic, caffeic, and syringic acids, or the total amount of phenolic acids.
3. There were several significant interactions among the added compounds, where the synthesis of the compounds was significantly lower than one could expect from the additivity of positive effects.

In sum, one can divide these compounds into three differently behaving classes: the first class included ellagic, *o*-coumaric, *p*-coumaric, rosmarinic and isoferulic acids, the second comprised gallic, syringic, caffeic and protocatechuic acids, while the third class was represented by ferulic acid alone.

#### 3.1.4. The Effect of PGRs on the Antioxidant Potential and Total Phenolic Content of Biomass

##### Agar Microshoot Cultures

The CUPRAC, FRAP, DPPH and F-C methods were used to test agar microshoot cultures growing on media containing different concentrations of BA and NAA (4 variants), respectively (mg/L): 1 and 1, 1 and 2, 2 and 1, and for medium variants with 1 mg/L cytokinin and 1 mg/L auxin (2iP and NAA, KIN and IAA, KIN and IBA, Zea and IBA, Zea and NAA) (5 variants) cultured over 10, 20 and 30-day growth periods (Tables S13 and S14). The antioxidant potentials of samples differed from one another depending on the tested medium variant (Table 4 and Tables S13 and S14). Antioxidant activity of biomass extracts evaluated by the CUPRAC method ranged from 2.09 to 4.13 mmol TE/100 g DW. In the control sample, the antioxidant activity ranged from 1.50 to 2.10 mmol TE/100 g DW (Table 4 and Table S13). The highest antioxidant potential was confirmed for the medium containing 1 mg/L KIN and 1 mg/L IAA after a 30-day growth period, and it was 2.0 times higher than in the control sample. The lowest antioxidant potential was recorded for the media containing 1 mg/L BA and 1 mg/L NAA, and also 2 mg/L BA and 1 mg/l NAA after a 10-day growth period (Table 4 and Tables S13 and S14).

Antioxidant activity of biomass extracts estimated with the FRAP method ranged from 0.52 to 1.42 mmol TE/100 g DW (Table 4 and Tables S13 and S14). The highest antioxidant potential was demonstrated for the medium variant containing 2 mg/L BA and 1 mg/L NAA after a 30-day growth period. The lowest antioxidant potential was confirmed for the variants containing 1 mg/L 2iP and 1 mg/L NAA after 10 days of culture (Table 4 and Tables S13 and S14). Antioxidant activity of biomass extracts evaluated by the DPPH method ranged from 0.49 to 30.89 mmol TE/100 g DW (Table 4 and Tables S13 and S14). The highest antioxidant potential was demonstrated for the medium variant containing 1 mg/L KIN and 1 mg/L IAA after a 30-day growth period, the lowest for the variant containing 2 mg/L BA and 1 mg/L NAA after 20 days of culture (Table 4 and Tables S13 and S14). The polyphenol content of biomass extracts estimated using the F-C method ranged from 2.42 to 8.69 mmol TE/100 g DW (Table 4 and Tables S13 and S14). The highest polyphenol content (8.69 mmol TE/100 g DW) was demonstrated for microshoots cultured on the medium variant with 1 mg/L Zea and 1 mg/L NAA collected after 10 days of growth. The lowest polyphenol content (2.42 mmol TE/100 g DW) was documented for the variant containing 2 mg/L BA and 1 mg/L NAA after 30 days of culture (Table 4 and Tables S13 and S14).

##### Agitated Microshoot Cultures

In agitated microshoot cultures, the antioxidant potential of biomass from six variants of the MS medium, containing PGRs at a concentration of 1 mg/L each (BA and NAA, 2iP and NAA, KIN and IAA, KIN and IBA, Zea and IBA, Zea and NAA) and cultured for periods of 10 and 20 days was evaluated. The antioxidant potentials of samples differed from one another depending on the medium tested (Table 4 and Table S15). The antioxidant activity of biomass extracts evaluated with the CUPRAC method ranged from 2.52 to 5.26 mmol TE/100 g DW (Table 4 and Table S15). The highest antioxidant potential (5.26 mmol TE/100 g DW) was demonstrated for the medium variant containing 1 mg/L KIN and 1 mg/L IAA after 20 days of culture, the lowest (2.52 mmol TE/100 g DW) for the variant containing 1 mg/L BA and 1 mg/L NAA after a 20-day growth period (Table 4 and Table S15).

The antioxidant activity of biomass extracts estimated with the FRAP method ranged from 0.51 to 1.26 mmol TE/100 g DW (Table 4 and Table S15). The highest antioxidant potential (1.26 mmol TE/100 g DW) was confirmed for the medium variant containing 1 mg/L KIN and 1 mg/L IAA after 20 days of culture. The lowest antioxidant potential (0.51 mmol TE/100 g DW) was documented for the medium variants containing 1 mg/L BA and 1 mg/L NAA after a 20-day growth period (Table 4 and Table S15). The antioxidant activity of biomass extracts tested with the DPPH method ranged from 14.55 to 25.95 mmol TE/100 g DW (Table 4 and Table S15). The highest antioxidant potential (25.95 mmol TE/100 g DW) was confirmed for the medium variant containing 1 mg/L KIN and 1 mg/L IAA after 20 days of culture. The lowest antioxidant potential (14.55 mmol TE/100 g DW) was documented for the media containing 1 mg/L KIN and 1 mg/L IAA after a 10-day growth period (Table 4 and Table S15).

The polyphenol content of biomass extracts estimated with the F-C method ranged from 2.49 to 5.24 mmol TE/100 g DW (Table 4 and Table S15). The highest polyphenol content (5.24 mmol TE/100 g DW) was demonstrated for the medium variant containing 1 mg/L KIN and 1 mg/L IAA after 20 days of culture. The lowest polyphenol content (2.49 mmol TE/100 g DW) was confirmed for the medium variants containing 1 mg/L 2iP and 1 mg/L NAA after a 20-day growth period (Table 4 and Table S15).

##### Statistical Analysis

Analyzing the combined datasets (Tables S4 and S5), one can conclude that IAA, NAA and IBA caused significant lowering of antioxidant activity in all cases except CUPRAC. Zea and 2iP always increased this property, and KIN did so in all cases except CUPRAC. The only interaction between two compounds (BA and NAA) that increased antioxidant activity was reported for the FRAP and F-C methods. Several interactions between time and compounds could be observed, but there were no strict rules.

### 3.2. Parent Plant Material

Quantitative analysis of glucosinolates in the *N. officinale* herb extracts was performed spectrophotometrically. The total glucosinolate content was 499.89 mg/100 g DW (Table 5). Of the 27 compounds analyzed, seven phenolic acids were detected in the *N. officinale* (caffeic, *o*-coumaric, *p*-coumaric, ferulic, gallic, rosmarinic and syringic) (Figure S6). The content of the individual compounds varied within a considerable range from 0.48 to 61.27 mg/100 g DW. The dominant metabolites were as follows: rosmarinic acid (61.27 mg/100 g DW), ferulic acid (14.32 mg/100 g DW) and gallic acid (13.67 mg/100 g DW). In the herb extracts the total phenolic acids content was 99.84 mg/100 g DW (Table 5). The antioxidant potential estimated by the CUPRAC method was 4.45 mmol TE/100 g DW (Table 5). Antioxidant activity determined by the FRAP assay was 0.76 mmol TE/100 g DW (Table 5). For the DPPH method, the antioxidant activity of the herb extract was 26.32 mmol TE/100 g DW (Table 5). In the F-C method, the total polyphenols content of the herb extract was 2.70 mmol TE/100 g DW (Table 5).

## 4. Discussion

The study documented for the first time the optimization of in vitro growing conditions for *N. officinale* microshoots. The experiments involved comprehensive optimization of culture conditions containing various PGR compositions, growth periods and types of in vitro cultures (agar, agitated). Moreover, the antioxidant potential of the studied biomass was evaluated. The experiments included both parent plant material and in vitro propagated microshoots. The research focused on testing the biomass response and incremental growth, and, moreover, the influence of various factors on the accumulation of secondary, biologically active metabolites such as glucosinolates and phenolic acids.

Significant effects of the PGRs, the growth period and type of culture were found on biomass increments in *N. officinale* in vitro cultures. Among the agar cultures, the highest Gi factor (Gi = 5.01) was obtained for the MS variant supplemented with 1 mg/L BA and 1 mg/L NAA after 30 days, and it was about 1.5 times higher than in the control sample (Table 1 and Table S2). By comparison, larger biomass increases were obtained for the agitated cultures. The highest biomass increment (Gi = 10.48) for this type of culture was recorded for the MS medium variant supplemented with 1 mg/L Zea and 1 mg/L NAA after a 20-day growth period (Table 1 and Table S2). In comparison with other microshoot cultures, the value of the Gi for the agar cultures was smaller than in *Schisandra chinensis* cv. Sadova agar cultures (max. 8.7, 60 days, MS medium containing 3 mg/L BA and 1 mg/L NAA). However, the *N. officinale* agitated in vitro cultures were characterized by greater increments in a shorter growth time. After a 20-day growth cycle, the increments were about 1.4 times greater than in *S. chinensis* cv. Sadova agitated cultures after a 60-day growth cycle [38].

The biotechnological experiments performed in our study proved the impact of in vitro culture conditions on the total amounts of glucosinolates. For the agar microshoot cultures, the highest content (194.77 mg/100 g DW) was obtained on the MS medium supplemented with 1 mg/L KIN and IAA after 30 days; it was 1.7 times higher than the highest content of the control set and 2.6 times lower than in the parent material (Figure 2, Table 5). In the agitated cultures, the total glucosinolate content was similar to that of the agar cultures. The highest total amount of glucosinolates (182.80 mg/100 g DW) in the agitated cultures was detected on the MS medium containing 1 mg/L BA and 1 mg/L NAA after 10 days of culture, and it was 2.7 times lower than in the parent plant (Figure 3, Table 5).

The *N. officinale* species was the object of studies by Rubin et al. [39]. They analyzed the amounts of only two glucosinolates—gluconasturtiin and glucotropaeolin, in methanolic extracts from the *N. officinale* herb and from in vitro agar shoot cultures grown on an MS agar medium without PGRs, containing 30 g/L sucrose and 4 g/L agar, using the HPLC method. In the parent plant material analyzed, the total amount of these two glucosinolates was 32.0 mg/100 g fresh weight (FW), and it was over 15 times lower compared to our total amount of glucosinolates, which was analyzed in a dry biomass of the *N. officinale* herb. In the study by Rubin et al., [39] these extracts from a fresh biomass of in vitro cultures were quantified as containing an amount 1.6 times higher (189.4 mg/100 g FW) compared with the total glucosinolate content of our agar microshoot cultures grown on the MS medium agar without PGRs (116.2 mg/100 g DW) [39]. The total amount of those two glucosinolates was comparable to our result obtained in agar cultures grown on the variant containing 1 mg/L KIN and 1 mg/L IAA collected after a 30-day growth period (194.77 mg/100 g DW) (Figure 2) [39].

In another study, the identification and quantification of glucosinolates was carried out using the liquid chromatography–mass spectrometry (LC-MS/MS) method [40]. In methanolic extracts from *N. officinale*, five glucosinolates were identified: gluconasturtiin, glucobrassicin, 4-hydroxyglucobrasscin, 4-methoxyglucobrassicin and glucoibarin. The major compound was gluconasturtiin (176.00 mg/100 g DW). The total glucosinolate content (279.00 mg/100 g DW) obtained in that study was almost two times lower in comparison with our results (499.89 mg/100 g DW) [40]. The *N. officinale* herb was also the object of research based on spectrophotometric analyses of total glucosinolate content [29]. The plant material consisted of the herb harvested at intervals of about one month. The results obtained with this method ranged from 592.21 to 1752.80 mg/100 g DW, and were respectively 1.2 times higher and 3.5 times higher in comparison with our analysis of *N. officinale* (499.89 mg/100 g DW) (Table 5) [29]. Moreover, in their study, Gallaher et al. also carried out analyses of another species from the *Brassicaceae* family—*Brassica oleracea* var. *italica* and *B. oleracea* var. *capitata* herbs harvested at intervals of about one month. The total amounts of glucosinolates obtained in extracts from *B. oleracea* var. *italica* ranged from 429.20 to 914.10 mg/100 g DW, and were respectively 1.2 times lower and 1.8 times higher in comparison with our analysis of *N. officinale* (499.89 mg/100 g DW) (Table 5). In *B. oleracea* var. *capitata* extracts, the total amounts of glucosinolates varied from 103.30 to 1033.30 mg/100 g DW, and were respectively 4.8 times lower and 2.1 times higher in comparison with our results (Table 5) [29]. In plants growing in vivo, many factors affect the production of secondary metabolites. Different contents may depend on, for example, climate, weather conditions, soil type, as well as storage of plant material [41].

Our study proved the influence of the applied variants of the MS medium and types of culture on the production of phenolic acids. The amounts of individual compounds were dependent on the PGRs used as well as on their concentration in the media, type of culture and duration of the growth period. The qualitative composition of the phenolic acids fraction was the same for the agar and agitated in vitro cultures (Table 2 and Table 3 and Tables S6–S12). In both cultures, we also obtained similar maximum total amounts of phenolic acids (237.52 and 236.74 mg/100 g DW, respectively). The predominant phenolic acids in both cultures were protocatechuic and gallic acids (Table 2 and Table 3 and Tables S6–S12). For the agar microshoot cultures, the highest amount of phenolic acids (237.52 mg/100 g DW) was 4.3 times higher than in the control cultures (55.76 mg/100 g DW) (Table 2 and Table 3 and Tables S6–S12). However, there were noticeable differences between the extracts from the in vitro cultures and those from the parent plant material in the qualitative and quantitative composition of the estimated compounds. In the microshoot cultures, ten phenolic acids (caffeic, *o*-coumaric, *p*-coumaric, ellagic, ferulic, gallic, isoferulic, protocatechuic, rosmarinic and syringic) were confirmed, while the parent plant material was found to contain seven phenolic acids (caffeic, *o*-coumaric, *p*-coumaric, ferulic, gallic, rosmarinic and syringic acids) (Table 2, Table 3 and Table 5). The maximum total amount of phenolic acids in extracts from the agar and agitated in vitro cultures was about 2.4 times higher than in the herb (99.84 mg/100 g DW) (Table 2, Table 3 and Table 5).

Earlier, Boligon et al. [15], using the HPLC-DAD method, had analyzed the qualitative composition of phenolic acids in crude extracts from purchased leaves of *N. officinale* grown in special hydroponic systems in Santa Maria in Brazil. Only caffeic and chlorogenic acids were found to be present in those extracts. Our HPLC-DAD analyses of parent plant extracts also detected caffeic acid, but chlorogenic acid was absent. In addition to caffeic acid, our extracts proved to contain six other phenolic acids (Table 5) [15]. Aires et al. [42] performed qualitative and quantitative phenolic acid estimations in methanol: water (*v*/*v*) extracts from young baby-leaves of *N. officinale* grown by organic farmers in the northern region of Portugal. The study revealed the presence of five phenolic acids: caffeic, chlorogenic, dicaffeoyltartaric and gallic acids. The main compounds were: dicaffeoyltartaric acid (5.50 mg/100 g DW) and chlorogenic acid (3.30 mg/100 g DW), which were not found in the herb extracts in our study (Table 5). The amounts of caffeic acid (0.20 mg/100 g DW) and gallic acid (1.60 mg/100 g DW) were respectively about 35.5 and 8.5 times lower in comparison with our results (Table 5). In another study, carried out by Zeb [43], seven phenolic acids were identified: caffeoylmalic, caftaric, *p*-coumaric, gallic, *p*-hydroxybenzoic, sinapic and vanillic, and also derivatives of coumaric, *p*-coumaric, ferulic and gallic acids in methanol: water (60%:40%) extracts from the leaves of wild *N. officinale* plants found in Pakistan. By comparison, our herb extracts also contained p-coumaric acid and gallic acid, but we did not confirm the presence of caftaric, *p*-hydroxybenzoic, sinapic and vanillic acids. In our study, we did not test for caffeoylmalic acid (Table 5) [43].

A study conducted by Taveira et al. [44] dealt with estimation of phenolic acids in biomass extracts from other species of the *Brassicaceae* family—*Brassica oleracea* L. var. *costata* DC. The study involved a qualitative analysis of phenolic acids in crude aqueous extracts from the biomass of shoot cultures grown on an MS medium agar containing 20 g/L sucrose and 2 mg/L BA, 0.1 mg/L NAA, and 0.5 µM gibberellic acid (GA). The composition of phenolic acids in those cultures differed from the composition of our *N. officinale* microshoot cultures. The acids identified in those extracts were chlorogenic acid and its derivatives such as: 4-acylchlorogenic, 3-caffeoylquinic, 3-*p*-coumarylquinic, 3-ferulylquinic and quinic acids [44]. Production of phenolic acids by in vitro cultures was also the subject of our earlier studies [45]. We had analyzed qualitative and quantitative composition of phenolic acids in extracts from *S. chinensis* cv. Sadova agar and agitated microshoot cultures grown on the MS medium with different PGRs of different concentrations. In methanolic extracts from these cultures we estimated eight phenolic acids: chlorogenic, cryptochlorogenic, gallic, neochlorogenic, protocatechuic, salicylic, syringic and vanillic acids. Similarly, the amounts of individual compounds and the total amount of phenolic acids depended on the type of in vitro culture and the variants of the applied media. The maximum total amount of phenolic acids in extracts from *S. chinensis* cv. Sadova agar microshoot cultures (357.93 mg/100 g DW) had been obtained by us for the cultures maintained on the MS medium containing 2 mg/L BA and 0.5 mg/L NAA, collected after 30 days of culture, and this amount was 1.5-fold higher than the maximum total content determined for the *N. officinale* agar microshoot cultures (237.52 mg/100 g DW) (Table 2). In extracts from agitated microshoot cultures of *S. chinensis* cv. Sadova, the maximum total content of phenolic acids (269.73 mg/100 g DW) was confirmed for extracts of the biomass cultured in the MS medium with 2 mg/L BA and 2 mg/L NAA over a 30-day growth period, and it was similar to the maximum total content determined in the *N. officinale* agitated microshoot cultures (236.74 mg/100 g DW) (Table 3) [45]. Quantitative differences in terms of phenolic acid yields were also found among the agitated shoot cultures of three cultivars of St John’s-wort—*Hypericum perforatum* cvs. Elixir, Helos, and Topas, cultured on variants of the MS medium differing in the composition of PGRs and their concentrations [46]. Extracts of all three cultivars were proved to produce the same phenolic acids: chlorogenic, 3,4-dihydroxyphenylacetic, *p*-coumaric, neo-chlorogenic, protocatechuic and syringic acids. However, the amounts of individual compounds and the total amount of phenolic acids depended on the cultivar studied and the applied media variants. The cultivar ‘Helos’, which had been grown on the MS medium containing 0.1 mg/L BA and 0.1 mg/L NAA for three-week culture cycles, was the richest source of phenolic acids [46]. The maximum total amount of phenolic acids in extracts from *H. perforatum* cv. ‘Helos’ agitated microshoot cultures (220.63 mg/100 g DW) was similar to the content in extracts from our *N. officinale* agitated microshoot cultures (236.74 mg/100 g DW). However, there were also differences in the concentrations of PGRs at which the maximum total amount of these compounds was obtained (Table 3) [46].

In our study, we evaluated the antioxidant capacity of the *N. officinale* parent plant material as well as the samples from in vitro microshoot cultures using the CUPRAC, FRAP and DPPH methods. Additionally, total phenolics (F-C) were estimated. The results were expressed as mmol TE/100 g DW. Our results for *N. officinale* microshoot cultures showed that the PGRs, duration of the growth period and the type of in vitro culture slightly influenced antioxidant potential. The maximal antioxidant potential using CUPRAC and DPPH method for extracts of agar microshoot cultures was respectively 2.0 and 9.7 times higher in comparison to maximal potential of control sample. In FRAP method the results were similar (Table 4 and Table S13). In extracts of agitated microshoot cultures the maximal antioxidant potential for CUPRAC method was 1.3 higher and for DPPH method 1.2 lower than in extracts of agar microshoot cultures (Table 4). The maximal potential of extracts from both types of cultures measured by CUPRAC and DPPH methods was obtained for MS medium containing 1 mg/L KIN and 1 mg/L IAA after 20 days in agitated cultures and 30 days in agar cultures. In comparison to agar control culture in agitated cultures was obtained 2.5 and 8.2 higher antioxidant potential respectively for CUPRAC and DPPH method. In FRAP method the maximal results were similar (Table 4 and Table S13). In extracts of parent plant antioxidant potential compared to maximal amounts obtained in agar and agitated microshoot cultures was 1.2 times lower in CUPRAC and DPPH method and 1.9 times lower in FRAP method (Table 4 and Table 5). In the F-C method, the maximal amount of phenolics in extracts of agar cultures was respectively 1.4 and 1.7 times higher than in maximal amount of extracts for control and agitated microshoot cultures and 3.2 times higher than in extracts form parent plant (Table 4 and Table 5 and Table S13). In extracts from agitated microshoot cultures the maximal amount of phenolic obtained in F-C method was 1.2 times lower and 1.9 times higher than in extracts of respectively agar control and parent plant material. (Table 4 and Table 5 and Table S13).

## 5. Conclusions

In this study, the initiation and optimization of the conditions for *N. officinale* in vitro cultures were performed for the first time. We assessed the influence of the qualitative and quantitative composition of different auxins and cytokinins in the MS culture medium, the type of culture, and the duration of the growth cycle on biomass growth; we also estimated the production of glucosinolates and phenolic acids, and the antioxidant activity of *N. officinale* microshoot cultures. We optimized the studied processes and therefore we can propose the best culture type and conditions for maintaining *N. officinale* cultures in vitro. Considering the finest biomass growth, optimal glucosinolates and phenolic acids production, were obtained in agitated cultures grown over 10-day periods on the MS medium containing 1 mg/L BA and 1 mg/L NAA. Due to high biomass increases, the best conditions for the production of phenolic acids were the agar cultures grown over 20-day periods on the same MS medium variant. For both types of cultures high antioxidant potential was proved for microshoots maintained on MS medium containing 1 mg/L KIN and 1 mg/L IAA. The studies have also shown that in agitated in vitro cultures, liquid medium and aeration of microshoots are the promoting conditions for microshoot growth. That could be because these conditions are similar to natural environment of this species.

The in vitro microshoots exhibited very interesting differences in comparison with the parent plant and provided alternative sources for obtaining health-promoting compounds.

## Figures and Tables

**Table 1:** The maximal values of the Gi of tested agar and agitated microshoots cultured under different duration of growth periods and composition of cytokinins and auxins in the MS media variants. Values represent the mean (±SD) of three experiments (*n* = 5). Table is corresponding with Tables S2 and S3.

Type of Microshoot Cultures	Growth Cycles (days)	Min Gi	Max Gi	Increase (fold)	MS Medium Variants
**Agar**	10	0.05 ± 0.01	1.75 ± 0.03	35.00	1 mg/L BA and 1 mg/L NAA
	20	0.37 ± 0.04	3.79 ± 0.05	10.24	
	30	1.28 ± 0.04	5.05 ± 0.14	3.95	
**Agitated**	10	1.47 ± 0.02	5.29 ± 0.07	3.60	1 mg/L BA and 1 mg/L NAA1 1 mg/L KIN and 1 mg/L IBA
	20	1.59 ± 0.01	10.48 ± 0.08	6.59	1 mg/L Zea and 1 mg/L NAA

**Table 2:** The range of individual and total phenolic acid contents (mg/100 g DW ± SD) dependent on duration of growth periods and composition of cytokinins and auxins in the MS media variants in agar microshoot cultures. Values represent the mean (±SD) of five samples (*n* = 5). Table is corresponding with Tables S6–S11.

Phenolic Acids	Min Content	Max Content	Increase (fold)	Conditions for Max Content		
				Growth Cycle (days)	MS Medium Variant	Gi
Caffeic acid	0.04 ± 0.01	16.65 ± 1.59	416.25	20	2 mg/L BA and 1 mg/L NAA	2.96 ± 0.06
*o*-Coumaric acid	0.03 ± 0.01	17.34 ± 2.09	578.00	30	1 mg/L 2iP and 1 mg/L IPA	2.06 ± 0.03
*p*-Coumaric acid	2.14 ± 0.18	18.95 ± 2.11	8.86	20	1 mg/L KIN and 1 mg/L IPA	0.42 ± 0.01
Ellagic acid	2.79 ± 0.25	6.56 ± 0.74	2.35	30	1 mg/L 2iP	1.59 ± 0.05
Ferulic acid	1.64 ± 0.20	18.13 ± 1.13	11.05	10	1 mg/L KIN and 1 mg/L IAA	0.30 ± 0.02
Gallic acid	1.40 ± 0.13	61.03 ± 5.89	43.59	30	2 mg/l BA and 1 mg/L NAA	2.95 ± 0.04
Isoferulic acid	0.02 ± 0.01	6.01 ± 0.59	300.50	10	1 mg/L KIN and 1 mg/L IBA	0.23 ± 0.03
Protocatechuic acid	0.78 ± 0.08	138.40 ± 12.99	177.44	20	2 mg/L BA and 1 mg/L NAA	2.96 ± 0.06
Rosmarinic acid	0.07 ± 0.01	33.30 ± 3.67	475.71	20	1 mg/L 2iP and 1 mg/L NAA	1.59 ± 0.01
Syringic acid	1.65 ± 0.18	9.65 ± 0.89	5.85	20	1 mg/L BA and 1 mg/L NAA	3.79 ± 0.05
**Total content**	**15.89 ± 1.66**	**237.52 ± 22.56**	**14.95**	**20**	**2 mg/L BA** **and** **1 mg/L NAA**	**2.96 ± 0.06**

**Table 3:** The range of individual and total phenolic acids contents (mg/100 g DW ± SD) dependent on duration of growth periods and composition of cytokinins and auxins in the MS media variants in agitated microshoot cultures. Values represent the mean (±SD) of five samples (*n* = 5). Table is corresponding with Table S12.

Phenolic Acids	Min Content	Max Content	Increase (fold)	Conditions for Max Content		
				Growth Cycle (days)	MS Medium Variant	Gi
Caffeic acid	0.02 ± 0.01	15.30 ± 1.66	765.00	10	1 mg/L Zea and 1 mg/L NAA	1.75 ± 0.01
*o*-Coumaric acid	0.74 ± 0.66	14.91 ± 1.35	20.15	10	1 mg/L BA and 1 mg/L NAA	5.29 ± 0.07
*p*-Coumaric acid	4.84 ± 0.45	31.66 ± 2.98	6.54	10	1 mg/L BA and 1 mg/L NAA	5.29 ± 0.07
Ellagic acid	2.93 ± 0.33	8.12 ± 0.79	2.69	20	1 mg/L KIN and 1 mg/L IBA	7.51 ± 0.03
Ferulic acid	1.58 ± 1.64	38.44 ± 4.12	24.33	10	1 mg/L BA and 1 mg/L NAA	5.29 ± 0.07
Gallic acid	29.84 ± 0.28	53.34 ± 5.12	1.79	10	1 mg/L Zea and 1 mg/L NAA	1.75 ± 0.01
Isoferulic acid	0.02 ± 0.01	15.17 ± 1.49	758.50	10	1 mg/L BA and 1 mg/L NAA	5.29 ± 0.07
Protocatechuic acid	4.94 ± 0.48	132.26 ± 12.02	26.77	10	1 mg/L Zea and 1 mg/L NAA	1.75 ± 0.01
Rosmarinic acid	2.95 ± 0.33	36.34 ± 3.71	12.32	20	1 mg/L 2iP and 1 mg/L NAA	1.59 ± 0.01
Syringic acid	1.65 ± 0.18	21.50 ± 2.12	13.03	10	1 mg/L BA and 1 mg/L NAA	5.29 ± 0.07
**Total content**	**70.80 ± 7.12**	**236.74 ± 19.30**	**3.34**	**10**	**1 mg/L Zea** **and** **1 mg/L NAA**	**1.75 ± 0.01**

**Table 4:** The maximal antioxidant potential estimated by CUPRAC, FRAP and DPPH methods and total phenolic F-C method (expressed in mmol TE/100 g DW ± SD) dependent on duration of growth periods and composition of cytokinins and auxins in the MS media variants in microshoot cultures. Values represent the mean (±SD) of five samples (*n* = 5). Table corresponding with Tables S13–S15.

Culture Type	Method	Min (mmol TE/100 g DW ± SD)	Max (mmol TE/100 g DW ± SD)	Increase (fold)	Conditions for Max Antioxidant Parameters		
					Growth Cycle (days)	MS Medium Variant	Gi
**Agar**	CUPRAC	1.50 ± 0.19	4.13 ± 0.80	1.69	30	1 mg/L KIN and 1 mg/L IAA	2.39 ± 0.05
	FRAP	0.52 ± 0.02	1.42 ± 0.02	2.73	30	2 mg/L BA and 1 mg/L NAA	2.95 ± 0.04
	DPPH	0.49 ± 0.04	30.89 ± 1.82	63.04	30	1 mg/L KIN and 1 mg/L IAA	2.39 ± 0.05
	F-C	2.42 ± 0.10	8.69 ± 3.44	3.59	10	1 mg/L Zea and 1 mg/L NAA	3.27 ± 0.05
**Agitated**	CUPRAC	2.52 ± 0.15	5.26 ± 0.10	2.09	20	1 mg/L KIN and 1 mg/L IAA	8.08 ± 0.06
	FRAP	0.51 ± 0.04	1.26 ± 0.04	2.47	20	1 mg/L KIN and 1 mg/L IAA	8.08 ± 0.06
	DPPH	14.55 ± 0.96	25.95 ± 4.07	1.78	20	1 mg/L KIN and 1 mg/L IAA	8.08 ± 0.06
	F-C	2.49 ± 0.13	5.24 ± 0.20	2.10	20	1 mg/L KIN and 1 mg/L IAA	8.08 ± 0.06

**Table 5:** The contents (mg/100 g DW ± SD) of total glucosinolates and individual and total phenolic acids, antioxidant activity estimated by CUPRAC, FRAP, DPPH methods and total phenolics F-C method (expressed as TE in mmol /100 g DW ± SD) in the extracts from parent plant material.

Total Glucosinolate Content (mg/100g DW ± SD)	Phenolic Acid Contents (mg/100g DW ± SD)		Antioxidant Activity	
			Method	(mmol TE/100 g DW ± SD)
499.89 ± 34.59	Caffeic acid	7.10 ± 0.65	CUPRAC	4.45 ± 0.02
	*o*-Coumaric acid	2.39 ± 0.19		
	*p*-Coumaric acid	0.61 ± 0.05	FRAP	0.76 ± 0.08
	Ferulic acid	14.32 ± 1.43		
	Gallic acid	13.67 ± 1.12	DPPH	26.32 ± 8.23
	Rosmarinic acid	61.27 ± 5.87		
	Syringic acid	0.48 ± 0.05	F-C	2.70 ± 0.31
	**Total content**	**99.84 ± 9.65**		

## Supplementary Materials

The following are available online at https://www.mdpi.com/2218-273X/10/9/1216/s1, Figure S1: Morphological appearance of *N. officinale* agar microshoots grown over 10, 20 and 30 days growth periods on control MS medium without PGRs, and on different tested variants of MS medium containing: 1 mg/L BA, 1 mg/L 2iP, 1 mg/l KIN, 1 mg/L Zea, 1 mg/L BA and 1 mg/L NAA, 2 mg/L BA and 1 mg/L NAA, 1 mg/L BA and 2 mg/l NAA, Figure S2: Morphological appearance of *N. officinale* agar microshoots grown over 10 days growth periods on different variants of MS medium containing 1 mg/L of cytokinins: BA, 2iP, KIN, Zea, and 1 mg/L auxins: 2, 4-D, IAA, IBA, IPA, NAA, Figure S3: Morphological appearance of *N. officinale agar* microshoots grown over 20 days growth periods on different variants of MS agar media containing 1 mg/L of cytokinins: BA, 2iP, KIN, Zea, and 1 mg/L auxins: 2,4-D, IAA, IBA, IPA, NAA. Figure S4: Morphological appearance of *N. officinale* agar microshoots grown over 30 days growth periods on different variants of MS agar media containing 1 mg/l of cytokinins: BA, 2iP, KIN, Zea, and 1 mg/l auxins: 2,4-D, IAA, IBA, IPA, NAA. Figure S5: Morphological appearance of *N. officinale* agitated microshoots grown over 10 and 20 days growth periods on different variants of MS medium. Figure S6. The representative HPLC-UV chromatogram (λ = 254 nm) of methanolic extracts from *N. officinale,* agar (A) and agitated (B) microshoot cultures (MS medium containing 1 mg/L Zea and 1 mg/L NAA after 20 days of growth period), and of intact plant (C). Table S1: The tested variants of culture medium supplemented with various PGRs combinations in concentrations of 1 mg/l each cytokinin and auxin. Table S2: The values of growth index—Gi* ± SD of *N. officinale* from tested agar in vitro cultures with different growth cycles and MS variant medium. Table S3: The values of growth index—Gi of *N. officinale* from tested *agitated* in vitro cultures with different growth cycles and MS variant medium. Table S4: Significant coefficients of the fitted models, together with their absolute values. Table S5: Standardized significant coefficients of the fitted models, together with their absolute values. Table S6: The influence of selected cytokinins (without auxin) and MS media without PGRs (control) on phenolic acid contents (mg/100 g DW ± SD) in extracts from *N. officinale* agar microshoots cultivated under different duration of growth periods. Table S7: Phenolic acids contents (mg/100 g DW ± SD) in extracts from *N. officinale* agar microshoots cultivated under different duration of growth periods on MS media variants with different concentration of cytokinin—BA and auxin—NAA. Table S8: Phenolic acid contents (mg/100 g DW ± SD) in extracts from *N. officinale* agar microshoots cultivated under different duration of growth periods on MS media variants with cytokinin—BA (1 mg/L) and auxins: IAA, IBA, IPA and NAA (1 mg/L). Table S9: Phenolic acid contents (mg/100 g DW ± SD) in extracts from *N. officinale* agar microshoots cultivated under different duration of growth periods on MS media variants with cytokinin—2iP (1 mg/L) and auxins: IAA, IBA, IPA and NAA (1 mg/L). Table S10: Phenolic acid contents (mg/100 g DW ± SD) in extracts from *N. officinale* agar microshoots cultivated under different duration of growth periods on MS media variants with cytokinin—KIN (1 mg/L) and auxins: IAA, IBA, IPA and NAA (1 mg/L). Table S11: Phenolic acid contents (mg/100 g DW ± SD) in extracts from *N. officinale* agar microshoots cultivated under different duration of growth periods on MS media variants with cytokinin—Zea (1 mg/L) and auxins: IAA, IBA, IPA and NAA (1 mg/L). Values represent the mean (±SD) of five experiments (*n* = 5). Table S12: Phenolic acid contents (mg/100 g DW ± SD) in extracts from *N. officinale* agitated microshoots cultivated under different duration of growth periods on MS media variants with cytokinins—BA, 2iP, KIN, Zea (1 mg/L) and auxins: IAA, NAA and IBA (1 mg/L). Table S13: The antioxidant activity estimated by CUPRAC, FRAP, DPPH methods and total phenolic F-C method (expressed in mmol TE/100 g DW) of *N. officinale* agar in vitro cultures cultivated on different MS medium variants and growth cycles. Table S14: The antioxidant activity estimated by CUPRAC, FRAP, DPPH methods and total phenolic F-C method (expressed in mmol TE/100 g DW) of *N. officinale* agar in vitro cultures cultivated on different MS medium variants and growth cycles. Table S15: The antioxidant activity estimated by CUPRAC, FRAP, DPPH methods and total phenolic F-C method (expressed in mmol TE/100 g DW ± SD) of *N. officinale* agitated microshoot cultures cultivated on different duration of growth cycles and variants of MS media.
