# Supplementary material for: The Influence of Nasturtium officinale R. Br. Agar and Agitated Microshoot Culture Media on Glucosinolate and Phenolic Acid Production, and Antioxidant Activity
Source: Biomolecules. 2020 Aug 21;10(9):1216. doi: 10.3390/biom10091216 (PMC7565577; doi:10.3390/biom10091216)
Supplement: Supplementary file 1 [file biomolecules-10-01216-s001.pdf]

Supplementary Data

The Influence of *Nasturtium officinale* R. Br. Agar and Agitated Microshoot Culture Media on Glucosinolate and Phenolic Acid Production, and Antioxidant Activity

Marta Klimek-Szczykutowicz [1], Agnieszka Szopa [1,*], Michał Dziurka [2], Łukasz Komsta [3], Michał Tomczyk [4], and Halina Ekiert [1]

[1] Chair and Department of Pharmaceutical Botany, Jagiellonian University, Medical College, ul. Medyczna 9, 30-688 Kraków, Poland; marta.klimek-szczykutowicz@doctoral.uj.edu.pl (M.K.S.); a.szopa@uj.edu.pl (A.S.); halina.ekiert@uj.edu.pl (H.E.)

[2] Polish Academy of Sciences, The Franciszek Górski Institute of Plant Physiology, ul. Niezapominajek 21, 30-239 Kraków, Poland; m.dziurka@ifr-pan.edu.pl (M.D.)

[3] Department of Medicinal Chemistry, Faculty of Pharmacy with Division of Medical Analytics, Medical University of Lublin, ul. Chodźki 4a, 20-093 Lublin, Poland; lukasz.komsta@umlub.pl (Ł.K.)

[4] Department of Pharmacognosy, Faculty of Pharmacy with the Division of Laboratory Medicine, Medical University of Białystok, ul. Mickiewicza 2a, 15-230 Białystok, Poland; michal.tomczyk@umb.edu.pl (M.T.)

* Correspondence: a.szopa@uj.edu.pl; Tel.: +48-12-620-54-36 (A.S.)

MS medium variant	Growth period (days)		
	10	20	30
0 (control)	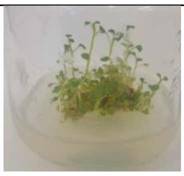	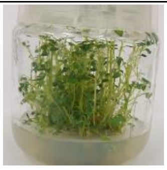	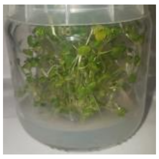
1 mg/l BA	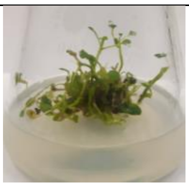	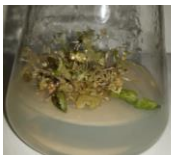	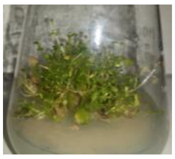
1 mg/l 2iP	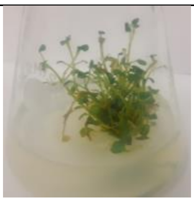	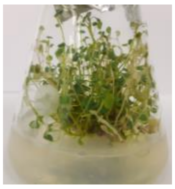	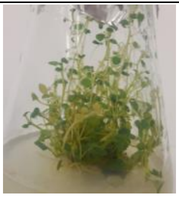
1 mg/l KIN	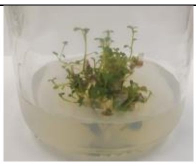	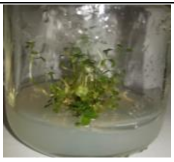	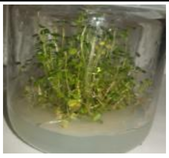
1 mg/l Zea	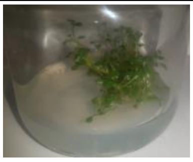	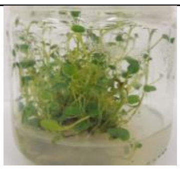	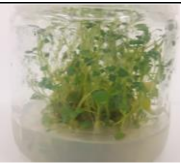
1 mg/l BA and 1 mg/l NAA	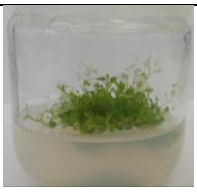	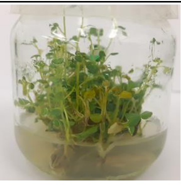	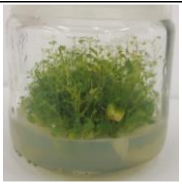
2 mg/l BA and 1 mg/l NAA	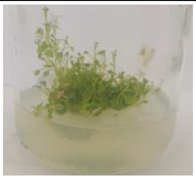	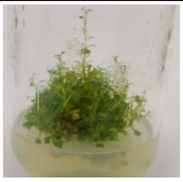	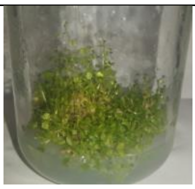
1 mg/l BA and 2 mg/l NAA	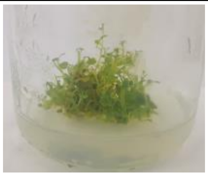	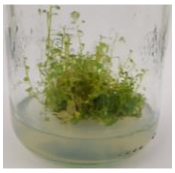	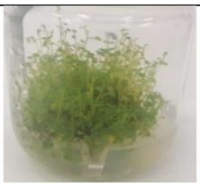

Figure S1. Morphological appearance of *N. officinale* agar microshoots grown over 10, 20 and 30 days growth periods on control MS medium without PGRs, and on different tested variants of MS medium containing: 1 mg/l BA, 1 mg/l 2iP, 1 mg/l KIN, 1 mg/l Zea, 1 mg/l BA and 1 mg/l NAA, 2 mg/l BA and 1 mg/l NAA, 1 mg/l BA and 2 mg/l NAA.

Auxins	Cytokinins			
	1 mg/l BA	1 mg/l 2iP	1 mg/l KIN	1 mg/l Zea
1 mg/l 2,4-D*	-	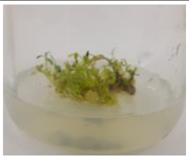	-	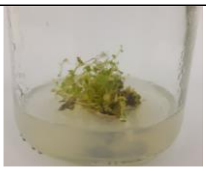
1 mg/l IAA	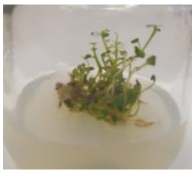	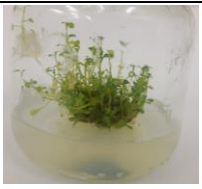	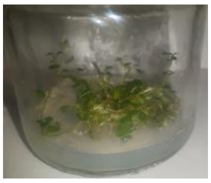	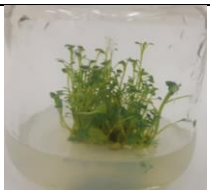
1 mg/l IBA	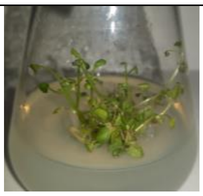	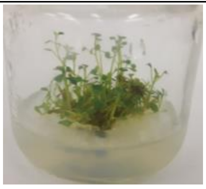	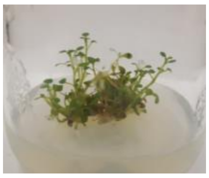	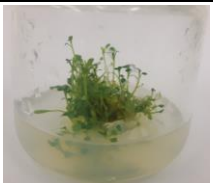
1 mg/l IPA	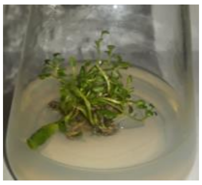	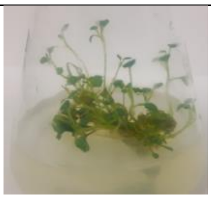	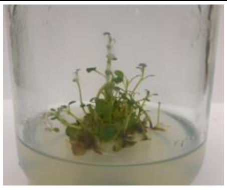	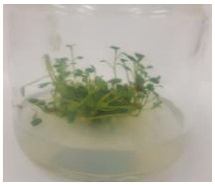
1 mg/l NAA	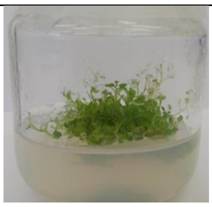	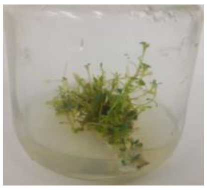	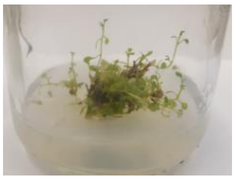	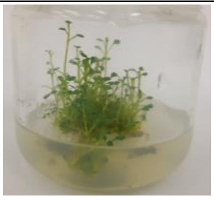

*on MS variants with 2,4-D the microshoot biomass was died

Figure S2. Morphological appearance of *N. officinale* agar microshoots grown over 10 days growth periods on different variants of MS medium containing 1 mg/l of cytokinins: BA, 2iP, KIN, Zea, and 1 mg/l auxins: 2,4-D, IAA, IBA, IPA, NAA.

Auxins	Cytokinins			
	1 mg/l BA	1 mg/l 2iP	1 mg/l KIN	1 mg/l Zea
1 mg/l 2,4-D*	-	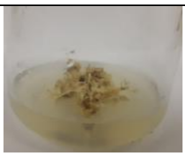	-	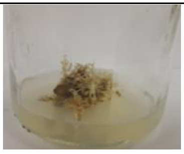
1 mg/l IAA	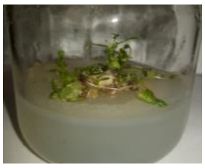	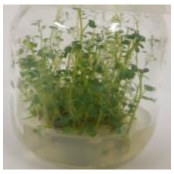	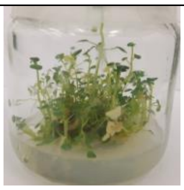	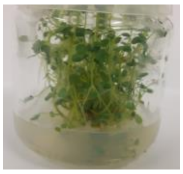
1 mg/l IBA	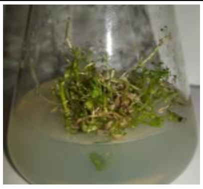	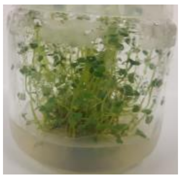	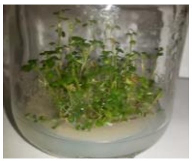	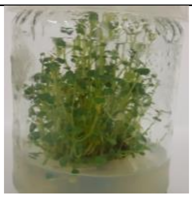
1 mg/l IPA	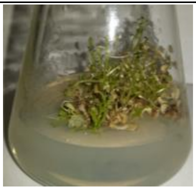	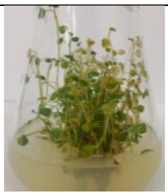	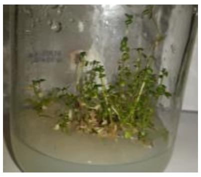	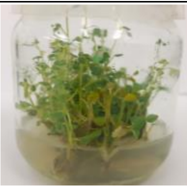
1 mg/l NAA	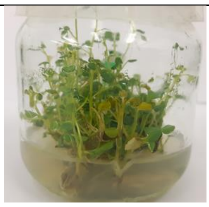	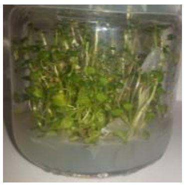	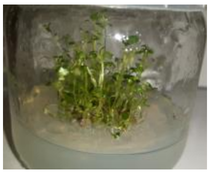	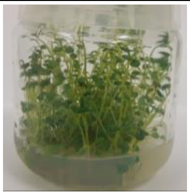

*on MS variants with 2,4-D the microshoot biomass was died

Figure S3. Morphological appearance of *N. officinale agar* microshoots grown over 20 days growth periods on different variants of MS agar media containing 1 mg/l of cytokinins: BA, 2iP, KIN, Zea, and 1 mg/l auxins: 2,4-D, IAA, IBA, IPA, NAA.

Auxins	Cytokinins			
	1 mg/l BA	1 mg/l 2iP	1 mg/l KIN	1 mg/l Zea
1 mg/l 2,4-D*	-	-	-	-
1 mg/l IAA	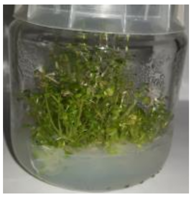	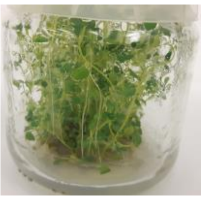	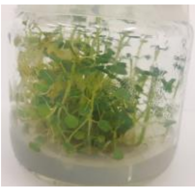	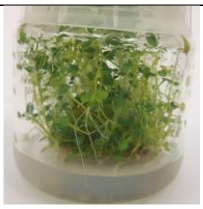
1 mg/l IBA	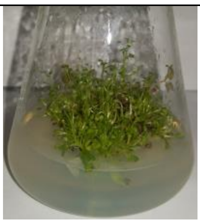	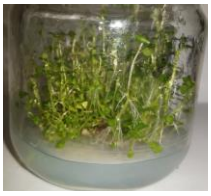	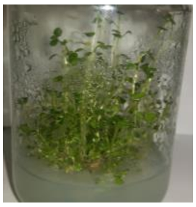	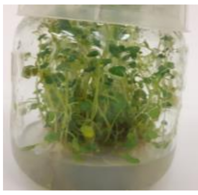
1 mg/l IPA	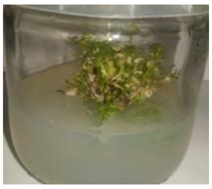	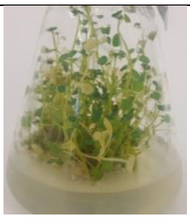	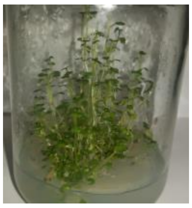	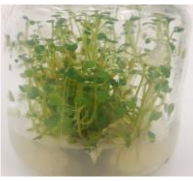
1 mg/l NAA	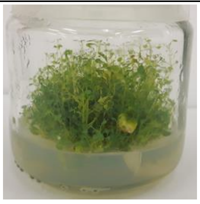	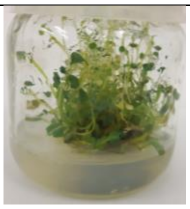	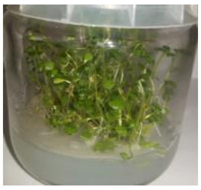	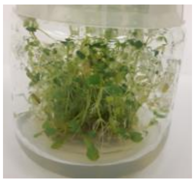

*on MS variants with 2,4-D the microshoot biomass was died

Figure S4. Morphological appearance of *N. officinale* agar microshoots grown over 30 days growth periods on different variants of MS agar media containing 1 mg/l of cytokinins: BA, 2iP, KIN, Zea, and 1 mg/l auxins: 2,4-D, IAA, IBA, IPA, NAA.

MS medium variant	Growth period (days)	
	10	20
1mg/l BA and 1 mg/l NAA	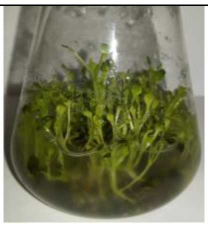	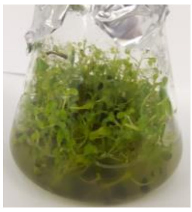
1 mg/l 2iP and 1 mg/l NAA	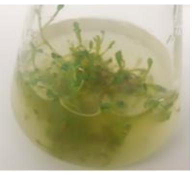	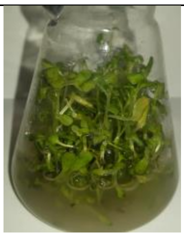
1 mg/l KIN and 1 mg/l IAA	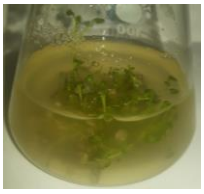	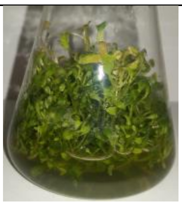
1 mg/l KIN and 1 mg/l IBA	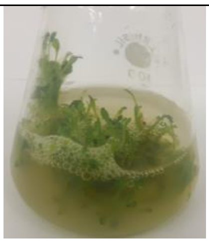	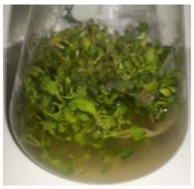
1 mg/l Zea and 1mg/l IBA	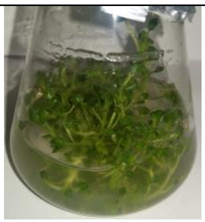	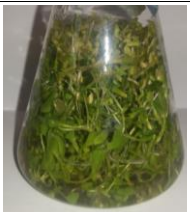
1 mg/l Zea and 1 mg/l NAA	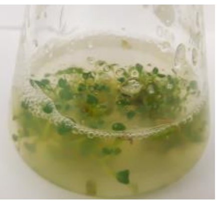	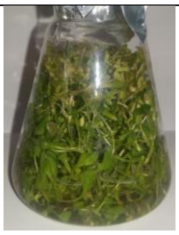

Figure S5. Morphological appearance of *N. officinale* agitated microshoots grown over 10 and 20 days growth periods on different variants of MS medium.

(A)

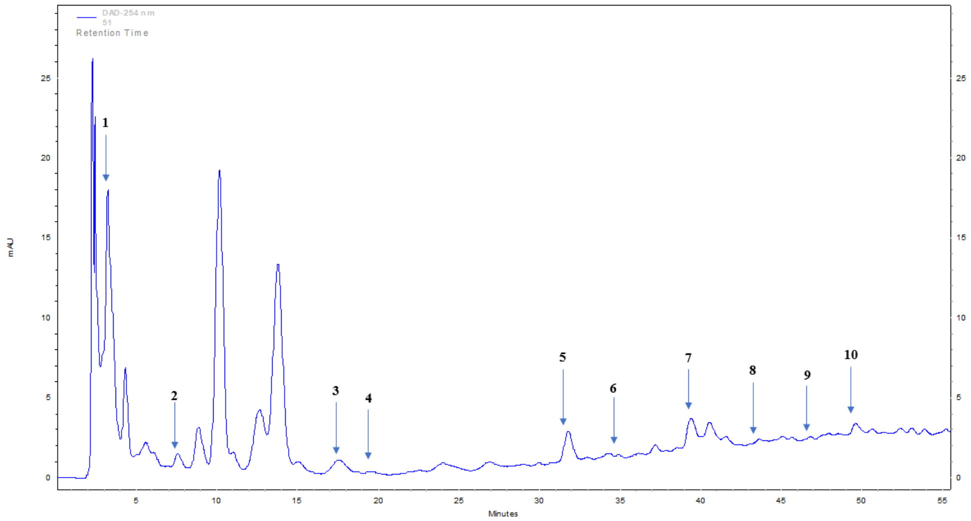

(B)

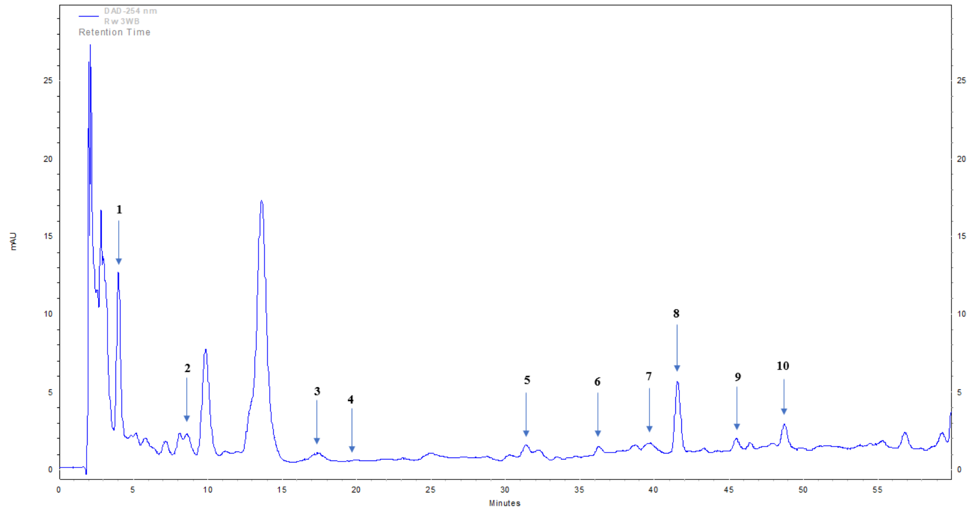

(C)

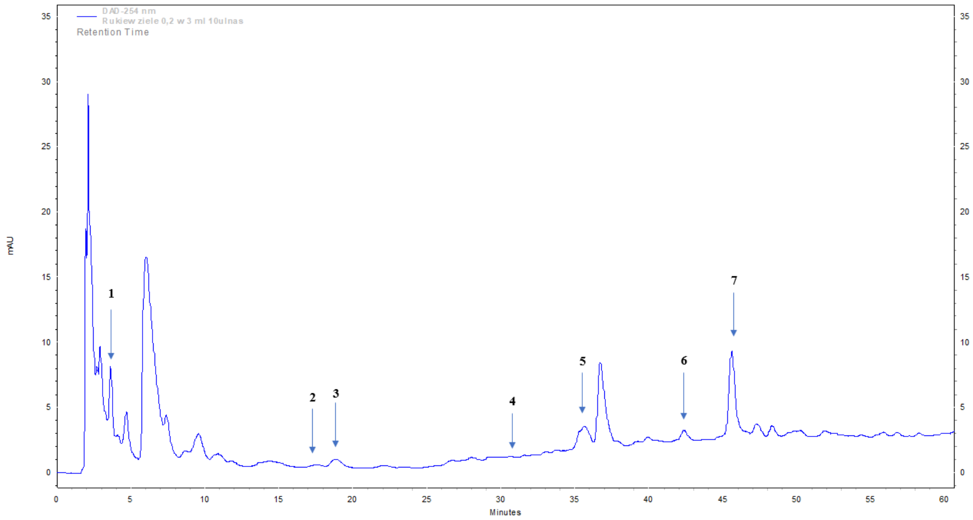

Figure S6. The representative HPLC-UV chromatogram (λ = 254 nm) of methanolic extracts from *N. officinale*, agar **(A)** and agitated **(B)** microshoot cultures (MS medium containing 1 mg/l Zea and 1 mg/l NAA after 20 days of growth period), and of intact plant **(C)**; 1- gallic acid, 2- protocatechuic acid, 3- caffeic acid, 4- syringic acid, 5- p-coumaric acid, 6- ferulic acid, 7- isoferulic acid, 8- o-couaric acid, 9- ellagic acid, 10 – rosmarinic acid.

Table S1. The tested variants of culture medium supplemented with various PGRs combinations in concentrations of 1 mg/l each cytokinin and auxin.

**Cytokinins**	**Auxins**
Zeatin (Zea)	3-Indoleacetic acid (IAA)
	2,4-Dichlorophenoxyacetic acid (2,4-D)
	1-Naphthaleneacetic acid (NAA)
	Indole-3-pyruvic acid (IPA)
	Indole-3-butyric acid (IBA)
6-(γ,γ-Dimethylallylamino)purine (2iP)	3-Indoleacetic acid (IAA)
	2,4-Dichlorophenoxyacetic acid (2,4-D)
	1-Naphthaleneacetic acid (NAA)
	Indole-3-pyruvic acid (IPA)
	Indole-3-butyric acid (IBA)
6-Benzyladenine (BA)	3-Indoleacetic acid (IAA)
	2,4-Dichlorophenoxyacetic acid (2,4-D)
	1-Naphthaleneacetic acid (NAA)
	Indole-3-pyruvic acid (IPA)
	Indole-3-butyric acid (IBA)
Kinetin (KIN)	3-Indoleacetic acid (IAA)
	2,4-Dichlorophenoxyacetic acid (2,4-D)
	1-Naphthaleneacetic acid (NAA)
	Indole-3-pyruvic acid (IPA)
	Indole-3-butyric acid (IBA)

Table S2. The values of growth index – Gi*±SD of *N. officinale* from tested agar *in vitro* cultures with different growth cycles and MS variant medium. Values represent the mean (±SD) of five experiments (n=5).

**MS medium variants**	**Growth cycle (days)**		
	**10**	**20**	**30**
0 (control)	0.15±0.04	3.26±0.11	1.97±0.04
1 mg/l BA	0.32±0.04	1.08±0.03	2.55±0.09
1 mg/l 2iP	0.50±0.01	1.35±0.03	1.59±0.05
1 mg/l KIN	0.05±0.01	0.37±0.04	1.28±0.04
1 mg/l Zea	0.25±0.01	2.01±0.04	2.06±0.04
2 mg/l BA and 1 mg/l NAA	1.17±0.03	2.96±0.06	2.95±0.04
1 mg/l BA and 2 mg/l NAA	0.76±0.03	1.84±0.05	2.64±0.08
1 mg/l BA and 1 mg/l NAA	1.75±0.03	3.79±0.05	5.05±0.14
1 mg/l BA and 1 mg/l IAA	0.49±0.01	0.76±0.03	4.10±0.07
1 mg/l BA and 1 mg/l IBA	0.39±0.02	1.05±0.03	2.01±0.10
1 mg/l BA and 1 mg/l IPA	0.46±0.03	1.18±0.05	1.50±0.02
1 mg/l 2iP and 1 mg/l IAA	0.38±0.03	2.06±0.06	2.48±0.04
1 mg/l 2iP and 1 mg/l IBA	0.62±0.04	1.52±0.02	2.42±0.02
1 mg/l 2iP and 1 mg/l IPA	0.18±0.02	1.26±0.05	2.06±0.03
1 mg/l 2iP and 1 mg/l NAA	0.35±0.01	1.59±0.01	3.13±0.05
1 mg/l KIN and 1 mg/l IAA	0.30±0.02	2.47±0.09	2.39±0.05
1 mg/l KIN and 1 mg/l IBA	0.23±0.03	1.20±0.02	3.01±0.01
1 mg/l KIN and 1 mg/l IPA	0.26±0.01	0.42±0.01	1.30±0.04
1 mg/l KIN and 1 mg/l NAA	0.07±0.01	0.89±0.05	2.84±0.08
1 mg/l Zea and 1 mg/l IAA	0.74±0.01	1.47±0.03	2.71±0.03
1 mg/l Zea and 1 mg/l IBA	0.64±0.01	2.03±0.04	3.36±0.03
1 mg/l Zea and 1 mg/l IPA	0.18±0.02	1.66±0.06	2.54±0.03
1 mg/l Zea and 1 mg/l NAA	0.69±0.04	2.23±0.03	3.27±0.05

* - calculated according to the formula: $Gi = [(Dw_1 - Dw_0)/Dw_0]$ where Dw_1 is the dry weight of microshoots at the end of the experiment and Dw_0 is the dry weight of the inoculum)

Table S3. The values of growth index – Gi of *N. officinale* from tested agitated *in vitro* cultures with different growth cycles and MS variant medium. Values represent the mean (±SD) of five experiments (n=5).

**MS medium variants**	**Growth cycle (days)**	
	**10**	**20**
1mg/l BA and 1 mg/l NAA	5.29±0.07	6.67±0.10
1 mg/l Zea and 1 mg/l NAA	1.75±0.01	10.48±0.08
1 mg/l Zea and 1 mg/l IBA	4.65±0.03	7.73±0.02
1 mg/l 2iP and 1 mg/l NAA	2.68±0.02	1.59±0.01
1 mg/l KIN and 1 mg/l IAA	1.47±0.02	8.08±0.06
1 mg/l KIN and 1 mg/l IBA	5.29±0.07	7.51±0.03

Table S4. Significant coefficients of the fitted models, together with their absolute values.

	GAL	PRO	CAF	SYR	COU	FER	ROS	IFE	ELA	OCU	TOT	CUPRAC	FRAP	DPPH	FOLIN	BIO	MOL
**(Intercept)**	-41.53	-	-	-	13.1	-	-	-	3.419	-	-98.18	21.05	8.999	131	40.75	-	9.802
**cs**	54.58	76.18	4.66	4.93	-	-	-	-	-	-	149	-	-	-	-	-	-
**t**	-	-	-	-	-0.3772	-	0.3568	-	-	-	-	-	-	-3.12	-	0.08887	-0.3246
**BA**	43.25	121.7	8.343	4.377	-11.19	-	-	-	-	-	167.4	4.734	-15.52	-	-54.26	-	-6.518
**NAA**	78.28	186.1	16.54	7.271	-	-	12.22	-	-	-	289.8	-	-13.92	-	-	-	-
**Zea**	64.98	73.39	11.05	5.307	-	14.16	-	-	-	-	200.9	12.01	12.64	129.8	66.8	-	-6.911
**IAA**	-	-	-	1.827	-	21.34	-	-	-	-	129.5	-	-13.23	-	-41.36	-	-7.642
**2iP**	65.31	67.36	-	7.368	-12.04	6.578	-	-	-	-5.005	147.4	12.26	12.58	130.1	45.21	-	-7.081
**KIN**	52.86	83.87	-	5.638	-10.92	-	-	-	-	-	136	-	12.09	146.8	42.98	-0.9538	-
**2,4-D**	-	-	-	-	-	-5.013	-	-	-	-	-	-	-	-	-	-	-
**IBA**	-	-	-	4.514	-	-	-	6.21	2.316	9.885	102.4	-	-14.88	-	-56.78	-	-
**BA:NAA**	-56.02	-105.4	-	-	-	-	-	-	-	-	-154.7	-	23.41	-	76.4	-	-
**BA:IAA**	-	-123.4	-	-	-	-21.5	-	-	-	-	-196.6	-	-	-	-	-	-
**Zea:NAA**	-82.44	-118.7	-6.877	-6.19	-	-10.49	-	-	-	-	-241.6	-	-	-	-	-	-
**2iP:NAA**	-77.94	-112.5	-17.6	-	-	-	-	-	-	-	-218.5	-	-	-	-	-	-
**KIN:NAA**	-83.42	-155	-19.56	-	-	-	-11.24	-	-	-	-240	-	-	-	-	-	-
**Zea:IAA**	-	-	-	-	-	-30.91	-	-	-	-	-154.1	-	-	-	-	-	-
**Zea:IBA**	-	-	-	-	-	-10.06	-	-	-1.667	-8.035	-	-	-	-	-	-	-
**2iP:IAA**	-	-	-	-	-	-25.43	-	-	-	-	-	-	-	-	-	-	-
**2iP:IBA**	-	-	-	-6.202	-	-4.426	-	-	-	-6.049	-	-	-	-	-	-	-
**cs:NAA**	-54.58	-76.18	-9.153	-4.93	-	-	-	-	-	-	-152.9	-	-	-	-	-	-
**t:BA**	-	-	-0.2933	-	0.5078	-	-	0.1261	-	-	-	-	-	-	-	-	0.3262
**t:NAA**	-	-2.296	-0.2709	-	-	-	-	-	-	-	-	-	-	-	-1.077	-	-
**t:Zea**	-	-	-	-	-	-0.4509	-	-	-	-	-	-	-	-	-	-	0.3366
**t:IAA**	-	-	-	-	-	-0.8552	-	-	-	-	-4.601	-	-	-	-	-	0.3968

**t:2iP**	-1.282	-	-	-	0.5799	-	-	0.1843	0.08637	0.4306	-	-	-	-	-	-	0.3651
**t:KIN**	-	-	-	-	0.659	-	-	-	-	-	-	-	-	-	-	-	-
**t:IBA**	-	-	-	-	-	-	-	-0.2592	-	-0.3458	-	-	-	-	-	-	-
**t:2iP:IAA**	-	-	-	-	-	1.056	-	-	-	-0.4442	-	-	-	-	-	-	-
**IPA**	-	-	-	1.46	5.997	-	-	-	-	-	-	-	-	-	-	-	-
**BA:IPA**	-	-	-	-	-	-	29.67	-	4.401	-	-	-	-	-	-	-	-
**BA:IBA**	-	-	-	-	-	-	-	-	-1.699	-8.72	-	-	-	-	-	-	-
**t:BA:IAA**	-	5.349	-	-	0.6992	0.9423	-	-	-	-	8.508	-	-	-	-	-	-
**t:BA:IPA**	-	-	-	-	-	-	-1.507	-	-0.279	-	-	-	-	-	-	-	-
**t:KIN:NAA**	-	-	-	-0.232	-	-	-	-	-	-	-	-	-	-	-	-	-
**t:2iP:NAA**	-	-	-	-	-	-	-	-	-	-0.3474	-	-	-	-	-	-	-
**t:Zea:IPA**	-	-	-	-	-	-0.4803	-	-	-	-	-	-	-	-	-	-	-
**t:2iP:IPA**	-	-	-	-	-	-0.5926	-	-	-0.2258	-	-	-	-	-	-	-	-
**t:Zea:IBA**	-	-	-	-	-	0.5146	-	-	-	-	-	-	-	-	-	-	-
**t:Zea:IAA**	-	-	-	-	-	1.484	-	-	-	-	-	-	-	-	-	-	-
**t:Zea:NAA**	-	-	-	-	-	0.6367	-	-	-	-	-	-	-	-	-	-	-
**cs:t**	-	-	-	-	-	-	-	-	-	-	-	-	-	-	-	0.3127	-
**BA:IBA**	-	-	-	-	-	-	-	-	-	-	-	-	-	-	-	-1.292	-
**cs:X2iP**	-	-	-	-	-	-	-	-	-	-	-	-	-	-	-	-1.689	-

Table S5. Standardized significant coefficients of the fitted models, together with their absolute values.

	GAL	PRO	CAF	SYR	COU	FER	ROS	IFE	ELA	OCU	TOT	CUPRAC	FRAP	DPPH	FOLIN	BIO	MOL
**(Intercept)**	0	-	-	-	0	-	-	-	0	-	0	0	0	0	0	-	0
**cs**	1.356	0.7444	0.4204	0.9255	-	-	-	-	-	-	1.026	-	-	-	-	-	-
**t**	-	-	-	-	-0.9543	-	0.4441	-	-	-	-	-	-	-0.2573	-	0.3555	-1.418
**BA**	1.633	1.807	1.144	1.249	-1.933	-	-	-	-	-	1.753	0.4098	-3.819	-	-2.37	-	-1.076
**NAA**	2.894	2.706	2.221	2.031	-	-	1.016	-	-	-	2.97	-	-3.032	-	-	-	-
**Zea**	1.844	0.819	1.139	1.138	-	1.59	-	-	-	-	1.58	0.6759	2.021	0.6025	1.897	-	-1.753
**IAA**	-	-	-	0.343	-	2.099	-	-	-	-	0.8921	-	-1.516	-	-0.8419	-	-1.56
**2iP**	1.813	0.7356	-	1.546	-1.529	0.7231	-	-	-	-0.7922	1.134	0.4947	1.442	0.4328	0.9203	-	-1.445
**KIN**	1.433	0.8942	-	1.155	-1.354	-	-	-	-	-	1.022	-	1.681	0.5922	1.061	-0.1999	-
**2,4-D**	-	-	-	-	-	-0.2636	-	-	-	-	-	-	-	-	-	-	-
**IBA**	-	-	-	0.8474	-	-	-	1.329	0.8764	1.4	0.7056	-	-2.244	-	-1.52	-	-
**BA:NAA**	-2.221	-1.644	-	-	-	-	-	-	-	-	-1.7	-	6.583	-	3.815	-	-
**BA:IAA**	-	-0.6448	-	-	-	-1.13	-	-	-	-	-0.7236	-	-	-	-	-	-
**Zea:NAA**	-1.095	-0.6202	-0.3316	-0.6211	-	-0.5516	-	-	-	-	-0.8896	-	-	-	-	-	-
**2iP:NAA**	-1.035	-0.5877	-0.8484	-	-	-	-	-	-	-	-0.8043	-	-	-	-	-	-
**KIN:NAA**	-1.108	-0.8094	-0.9431	-	-	-	-0.336	-	-	-	-0.8835	-	-	-	-	-	-
**Zea:IAA**	-	-	-	-	-	-1.626	-	-	-	-	-0.5672	-	-	-	-	-	-
**Zea:IBA**	-	-	-	-	-	-0.5288	-	-	-0.3372	-0.6084	-	-	-	-	-	-	-
**2iP:IAA**	-	-	-	-	-	-1.337	-	-	-	-	-	-	-	-	-	-	-
**2iP:IBA**	-	-	-	-0.6223	-	-0.2327	-	-	-	-0.458	-	-	-	-	-	-	-
**cs:NAA**	-1.713	-0.9402	-1.043	-1.169	-	-	-	-	-	-	-1.33	-	-	-	-	-	-
**t:BA**	-	-	-0.894	-	1.95	-	-	0.9117	-	-	-	-	-	-	-	-	1.174
**t:NAA**	-	-0.7388	-0.8047	-	-	-	-	-	-	-	-	-	-	-	-0.9172	-	-
**t:Zea**	-	-	-	-	-	-1.087	-	-	-	-	-	-	-	-	-	-	1.735
**t:IAA**	-	-	-	-	-	-1.842	-	-	-	-	-0.6939	-	-	-	-	-	1.605

**t:2iP**	-0.765	-	-	-	1.584	-	-	0.9476	0.7852	1.465	-	-	-	-	-	-	1.477
**t:KIN**	-	-	-	-	1.797	-	-	-	-	-	-	-	-	-	-	-	-
**t:IBA**	-	-	-	-	-	-	-	-1.215	-	-1.073	-	-	-	-	-	-	-
**t:2iP:IAA**	-	-	-	-	-	1.203	-	-	-	-0.7288	-	-	-	-	-	-	-
**IPA**	-	-	-	0.2741	0.6816	-	-	-	-	-	-	-	-	-	-	-	-
**BA:IPA**	-	-	-	-	-	-	0.8866	-	0.8901	-	-	-	-	-	-	-	-
**BA:IBA**	-	-	-	-	-	-	-	-	-0.3436	-0.6603	-	-	-	-	-	-	-
**t:BA:IAA**	-	0.6054	-	-	0.9204	1.074	-	-	-	-	0.6786	-	-	-	-	-	-
**t:BA:IPA**	-	-	-	-	-	-	-0.9754	-	-1.223	-	-	-	-	-	-	-	-
**t:KIN:NAA**	-	-	-	-0.5045	-	-	-	-	-	-	-	-	-	-	-	-	-
**t:2iP:NAA**	-	-	-	-	-	-	-	-	-	-0.5699	-	-	-	-	-	-	-
**t:Zea:IPA**	-	-	-	-	-	-0.5472	-	-	-	-	-	-	-	-	-	-	-
**t:2iP:IPA**	-	-	-	-	-	-0.6752	-	-	-0.9895	-	-	-	-	-	-	-	-
**t:Zea:IBA**	-	-	-	-	-	0.5863	-	-	-	-	-	-	-	-	-	-	-
**t:Zea:IAA**	-	-	-	-	-	1.691	-	-	-	-	-	-	-	-	-	-	-
**t:Zea:NAA**	-	-	-	-	-	0.7254	-	-	-	-	-	-	-	-	-	-	-
**cs:t**	-	-	-	-	-	-	-	-	-	-	-	-	-	-	-	0.874	-
**BA:IBA**	-	-	-	-	-	-	-	-	-	-	-	-	-	-	-	-0.1201	-
**cs:X2ip**	-	-	-	-	-	-	-	-	-	-	-	-	-	-	-	-0.129	-

Table S6. The influence of selected cytokinins (without auxin) and MS media without PGRs (control) on phenolic acid contents (mg/100 g DW±SD) in extracts from *N. officinale* agar microshoots cultivated under different duration of growth periods. Values represent the mean (± SD) of five experiments (n=5).

Phenolic acids	MS medium variants														
	0 (control)			1 mg/l BA			1 mg/l 2iP			1 mg/l KIN			1 mg/l Zea		
	Growth cycle (days)														
	10	20	30	10	20	30	10	20	30	10	20	30	10	20	30
Caffeic acid	0.20±0.01	4.21±0.31	4.46±0.51	1.59±0.14	0.49±0.05	2.10±0.18	1.94±0.21	2.00±0.19	3.49±0.29	0.08±0.01	0.17±0.02	2.46±0.28	4.01±0.41	3.84±0.34	1.31±0.12
o-Coumaric acid	0.97±0.07	0.13±0.01	0.26±0.02	0.18±0.02	0.55±0.06	3.18±0.29	1.58±0.14	0.29±0.03	11.03±1.14	0.84±0.07	0.81±0.08	1.55±0.13	0.60±0.05	0.27±0.03	2.75±0.31
p-Coumaric acid	11.85±1.03	2.84±0.32	2.81±0.21	3.98±0.39	2.14±0.18	6.57±0.56	3.70±0.32	4.28±0.44	6.02±0.63	6.44±0.59	4.04±0.42	13.52±1.43	6.16±0.59	4.75±0.46	3.52±0.36
Ellagic acid	4.40±0.35	4.08±0.31	3.82±0.41	2.93±0.22	2.91±0.35	5.21±0.47	4.30±0.39	4.13±0.39	6.56±0.74	3.41±0.33	3.41±0.32	4.39±0.38	3.94±0.43	6.04±0.55	5.38±0.48
Ferulic acid	2.48±0.15	2.52±0.13	3.90±0.04	2.35±0.21	2.73±0.32	6.03±0.58	9.37±0.82	8.62±0.91	12.14±1.23	2.15±0.19	4.26±0.39	5.51±0.52	11.77±1.12	8.93±0.84	4.79±0.55
Gallic acid	12.17±1.11	18.00±1.69	20.49±2.11	12.97±1.32	1.40±0.13	10.21±1.02	12.26±1.26	5.36±0.48	7.00±0.66	6.82±0.70	2.98±0.33	6.72±0.64	15.41±1.67	24.57±2.31	13.71±1.22
Isoferulic acid	1.94±0.16	0.78±0.09	0.73±0.06	0.69±0.06	0.13±0.01	3.91±0.35	0.91±0.08	0.64±0.05	5.32±0.53	1.66±0.18	0.79±0.07	2.58±0.26	1.36±0.12	1.88±0.21	1.82±0.17
Protocatechuic acid	6.00±0.55	8.13±0.76	10.84±1.30	20.07±2.12	0.78±0.08	2.83±0.27	6.83±0.75	1.44±0.13	2.02±0.19	14.80±1.34	2.65±0.23	3.35±0.28	14.14±1.43	4.88±0.48	3.08±0.32
Rosmarinic acid	5.95±0.49	9.17±0.87	4.05±0.38	1.46±0.13	2.32±0.20	25.65±2.63	1.52±0.13	0.07±0.01	22.05±1.30	0.41±0.04	4.62±0.51	5.37±0.49	1.36±0.14	11.16±1.22	7.35±0.68
Syringic acid	2.17±0.17	2.52±0.18	4.40±0.39	2.67±0.24	2.44±0.22	2.46±0.34	3.79±0.41	4.79±0.51	4.09±0.39	3.16±0.28	2.23±0.24	3.61±0.33	3.57±0.33	2.21±0.19	2.38±0.25
**Total content**	**48.13±3.97**	**52.38±4.87**	**55.76±5.22**	**48.89±5.01**	**15.89±1.66**	**68.15±6.12**	**46.21±4.11**	**31.62±3.11**	**79.72±8.02**	**39.76±3.68**	**25.94±2.87**	**49.04±5.11**	**62.32±5.98**	**68.54±7.02**	**46.09±4.55**

Table S7. Phenolic acids contents (mg/100 g DW±SD) in extracts from *N. officinale* agar microshoots cultivated under different duration of growth periods on MS media variants with different concentration of cytokinin – BA and auxin – NAA. Values represent the mean (±SD) of five experiments (n=5).

**Phenolic acids**	**MS medium variants**								
	**1 mg/l BA and 1 mg/l NAA**			**2 mg/l BA and 1 mg/l NAA**			**1 mg/l BA and 2 mg/l NAA**		
	**Growth cycle (days)**								
	**10**	**20**	**30**	**10**	**20**	**30**	**10**	**20**	**30**
Caffeic acid	9.51±0.91	12.79±1.35	0.04±0.01	12.95±1.31	16.65±1,59	0.05±0.01	11.92±1.12	16.18±1.54	0.04±0.01
o-Coumaric acid	1.23±0.11	4.20±0.39	0.48±0.05	0.46±0.04	4.31±0.39	4.07±0.39	0.30±0.02	3.45±0.31	3.46±0.31
p-Coumaric acid	3.80±0.29	12.56±1.22	7.32±0.69	6.38±0.62	8.38±0.78	12.65±1.17	6.97±0.72	6.16±0.53	11.73±1.43
Ellagic acid	4.17±0.33	4.90±0.42	3.78±0.42	3.74±0.41	5.01±0.48	5.02±0.52	3.70±0.29	4.53±0.41	4.95±0.45
Ferulic acid	1.64±0.20	4.97±0.51	2.16±0.18	1.88±0.15	3.49±0.27	4.42±0.45	1.99±0.16	2.89±0.18	4.08±0.33
Gallic acid	18.92±1.99	29.94±3.01	33.35±3.11	24.27±2.54	34.40±3.26	61.03±5.89	23.15±2.18	31.03±3.14	24.98±2.37
Isoferulic acid	4.12±0.45	5.76±0.53	2.15±0.19	2.58±0.22	4.80±0.43	5.48±0.43	2.74±0.25	4.40±0.37	4.62±0.51
Protocatechuic acid	101.21±10.21	104.49±10.15	33.08±3.26	92.88±9.12	138.40±12.99	55.23±5.35	73.19±6.98	136.38±12.96	59.33±6.02
Rosmarinic acid	10.64±1.13	15.65±1.64	8.66±0.79	6.21±0.58	13.21±1.30	14.48±1.33	0.86±0.09	10.81±1.13	13.52±1.52
Syringic acid	5.09±0.49	9.65±0.89	6.45±0.59	7.23±0.66	8.86±0.84	8.52±0.78	5.71±0.44	6.59±0.61	7.87±0.67
**Total content**	**160.32±15.40**	**204.91±20.35**	**97.48±9.65**	**158.78±16.11**	**237.52±22.56**	**170.95±16.82**	**130.53±12.45**	**222.42±20.65**	**134.58±12.98**

Table S8. Phenolic acid contents (mg/100 g DW±SD) in extracts from *N. officinale* agar microshoots cultivated under different duration of growth periods on MS media variants with cytokinin – BA (1 mg/l) and auxins: IAA, IBA, IPA and NAA (1 mg/l). Values represent the mean (±SD) of five experiments (n=5).

**Phenolic acids**	**MS medium variants**											
	**1 mg/l BA and 1mg/l IAA**			**1 mg/l BA and 1mg/l IBA**			**1 mg/l BA and 1mg/l IPA**			**1 mg/l BA and 1mg/l NAA**		
	**Growth cycle (days)**											
	**10**	**20**	**30**	**10**	**20**	**30**	**10**	**20**	**30**	**10**	**20**	**30**
Caffeic acid	1.14±0.15	2.10±0.16	3.51±0.29	0.24±0.02	3.44±0.29	1.33±0.12	0.59±0.06	0.47±0.05	2.35±0.21	9.51±0.91	12.79±1.35	0.04±0.01
o-Coumaric acid	0.89±0.11	1.86±0.17	2.79±0.31	1.92±0.15	0.38±0.04	2.55±0.21	5.20±0.47	0.03±0.01	0.31±0.27	1.23±0.11	4.20±0.39	0.48±0.05
p-Coumaric acid	2.73±0.33	5.05±0.47	9.27±0.89	7.70±0.81	2.48±0.22	4.89±0.53	6.73±0.55	3.92±0.33	7.26±0.66	3.80±0.29	12.56±1.22	7.32±0.69
Ellagic acid	2.92±0.31	5.37±0.49	5.00±0.47	3.52±0.33	2.79±0.25	4.00±0.41	5.62±0.46	2.84±0.25	2.99±0.30	4.17±0.33	4.90±0.42	3.78±0.42
Ferulic acid	1.83±0.21	7.53±0.69	5.68±0.55	4.40±0.39	2.86±0.27	4.61±0.35	4.29±0.39	3.30±0.27	4.18±0.42	1.64±0.20	4.97±0.51	2.16±0.18
Gallic acid	10.71±1.11	2.45±0.21	10.05±1.01	4.93±0.51	3.45±0.30	4.47±0.51	9.34±1.00	2.93±0.30	2.42±0.21	18.92±1.99	29.94±3.01	33.35±3.11
Isoferulic acid	0.54±0.04	3.66±0.42	3.89±0.35	2.38±0.19	0.38±0.04	0.37±0.04	2.19±0.15	0.52±0.44	0.55±0.06	4.12±0.45	5.76±0.53	2.15±0.19
Protocatechuic acid	7.30±0.68	3.07±0.29	24.84±2.56	10.17±1.09	3.99±0.41	7.26±0.72	8.57±0.91	1.78±0.11	3.23±0.29	101.21±10.21	104.49±10.15	33.08±3.26
Rosmarinic acid	0.24±0.02	17.61±1.88	3.49±0.32	1.41±0.13	2.38±0.19	4.47±0.41	24.21±2.43	1.69±0.14	2.08±0.18	10.64±1.13	15.65±1.64	8.66±0.79
Syringic acid	2.14±0.19	2.23±0.21	1.65±0.18	2.62±0.31	4.12±0.39	1.77±0.19	3.65±0.42	3.89±0.41	4.15±0.38	5.09±0.49	9.65±0.89	6.45±0.59
**Total content**	**30.47±3.08**	**50.94±5.11**	**70.16±6.88**	**39.28±4.01**	**26.24±2.54**	**35.72±0.36**	**70.39±6.89**	**21.36±1.90**	**29.51±2.68**	**160.32±15.40**	**204.91±20.35**	**97.48±9.65**

Table S9. Phenolic acid contents (mg/100 g DW±SD) in extracts from *N. officinale* agar microshoots cultivated under different duration of growth periods on MS media variants with cytokinin – 2iP (1 mg/l) and auxins: IAA, IBA, IPA and NAA (1 mg/l). Values represent the mean (±SD) of five experiments (n=5).

**Phenolic acids**	**MS medium variants**											
	**1 mg/l 2iP and 1mg/l IAA**			**1 mg/l 2iP and 1mg/l IBA**			**1 mg/l 2iP and 1mg/l IPA**			**1 mg/l 2iP and 1mg/l NAA**		
	**Growth cycle (days)**											
	**10**	**20**	**30**	**10**	**20**	**30**	**10**	**20**	**30**	**10**	**20**	**30**
Caffeic acid	6.99±0.74	1.03±0.11	4.33±0.44	5.42±0.48	0.76±0.08	3.68±0.42	3.63±0.33	2.00±0.19	0.71±0.06	1.31±0.12	2.64±0.23	4.02±0.37
o-Coumaric acid	1.36±0.12	1.05±0.12	0.70±0.06	0.25±0.02	1.53±0.13	2.09±0.23	2.36±0.13	0.86±0.07	17.34±2.09	0.41±0.03	4.33±0.31	2.16±0.17
p-Coumaric acid	3.22±0.29	5.60±0.46	5.82±0.55	4.87±0.52	6.45±0.54	6.37±0.59	8.20±0.76	5.16±0.52	8.68±0.78	3.74±0.28	7.17±0.65	5.33±0.47
Ellagic acid	5.70±0.53	4.87±0.43	4.10±0.37	4.02±0.37	4.52±0.34	5.80±0.64	4.82±0.43	4.25±0.04	4.11±0.36	5.40±0.52	7.54±0.61	6.00±0.53
Ferulic acid	6.92±0.61	9.76±0.89	13.84±1.42	7.59±0.68	6.92±0.72	10.05±1.12	10.64±1.11	8.27±0.67	5.05±0.43	6.58±0.66	5.23±0.47	9.36±0.99
Gallic acid	12.49±1.12	24.37±0.28	26.61±2.45	10.53±1.09	11.31±1.85	18.66±1.99	12.76±1.18	4.76±0.32	9.73±0.90	11.73±1.12	32.33±2.92	14.57±1.26
Isoferulic acid	1.82±0.17	0.92±0.09	1.85±0.21	0.25±0.03	1.04±0.12	2.49±0.18	2.50±0.18	0.71±0.07	1.01±0.11	0.40±0.04	6.04±0.64	4.35±0.36
Protocatechuic acid	39.07±3.77	12.45±0.14	9.85±0.93	29.31±3.11	7.95±0.83	13.14±1.22	8.77±0.78	2.60±0.24	1.17±0.11	58.91±0.56	17.05±1.85	10.23±1.09
Rosmarinic acid	3.87±0.42	2.70±0.25	1.24±0.09	0.82±0.08	3.81±0.36	3.74±0.43	2.88±0.24	0.91±0.09	4.96±0.41	2.12±0.19	33.30±3.67	3.67±0.32
Syringic acid	5.22±0.48	3.72±0.31	4.60±0.39	3.09±0.33	5.85±0.63	4.64±0.48	3.07±0.27	4.40±0.39	2.59±0.21	5.59±0.52	9.56±0.96	5.17±0.53
**Total content**	**86.66±8.53**	**66.46±5.98**	**72.93±6.98**	**66.15±6.02**	**50.14±5.11**	**70.65±7.11**	**59.64±6.02**	**33.92±3.03**	**55.34±5.09**	**96.18±9.34**	**125.19±11.87**	**64.85±6.12**

Table S10. Phenolic acid contents (mg/100 g DW±SD) in extracts from *N. officinale* agar microshoots cultivated under different duration of growth periods on MS media variants with cytokinin – KIN (1 mg/l) and auxins: IAA, IBA, IPA and NAA (1 mg/l). Values represent the mean (±SD) of five experiments (n=5).

**Phenolic acids**	**MS medium variants**											
	**1 mg/l KIN and 1mg/l IAA**			**1 mg/l KIN and 1mg/l IBA**			**1 mg/l KIN and 1mg/l IPA**			**1 mg/l KIN and 1mg/l NAA**		
	**Growth cycle (days)**											
	**10**	**20**	**30**	**10**	**20**	**30**	**10**	**20**	**30**	**10**	**20**	**30**
Caffeic acid	4.86±0.50	0.89±0.09	0.51±0.05	0.83±0.08	6.22±0.59	2.97±0.32	0.28±0.03	0.77±0.08	5.77±0.53	0.10±0.01	0.14±0.02	3.97±0.42
o-Coumaric acid	1.32±0.11	0.13±0.01	0.28±0.02	7.85±0.67	1.79±0.16	1.56±0.13	0.53±0.05	4.97±0.46	1.78±0.12	0.42±0.38	1.47±0.16	0.52±0.04
p-Coumaric acid	7.10±0.68	2.76±0.31	2.69±0.23	6.73±0.62	13.88±1.23	6.06±0.59	3.75±0.32	18.95±2.11	5.55±0.49	4.07±0.39	6.76±0.55	5.60±0.49
Ellagic acid	4.77±0.45	4.04±0.43	4.67±0.44	6.36±0.58	3.94±0.38	4.67±0.54	2.96±0.27	6.55±0.70	5.04±0.45	3.50±0.29	4.86±0.52	4.02±0.38
Ferulic acid	18.13±1.13	3.08±0.28	3.40±0.33	5.24±0.45	6.58±0.65	7.16±0.68	2.87±0.26	8.65±0.82	8.60±0.91	4.01±0.33	6.42±0.59	6.61±0.59
Gallic acid	16.61±1.73	23.24±2.12	21.26±2.13	22.48±2.43	10.83±1.11	10.35±1.09	12.35±1.22	2.45±0.21	9.36±0.88	5.09±0.48	8.52±0.82	14.41±1.32
Isoferulic acid	1.40±0.13	0.20±0.02	0.36±0.04	6.01±0.59	1.60±0.14	0.60±0.06	0.78±0.07	6.15±0.55	1.78±0.18	0.78±0.07	2.64±0.25	0.76±0.07
Protocatechuic acid	50.17±5.11	29.38±2.88	9.30±0.88	31.97±3.14	15.73±1.42	9.08±0.99	16.30±1.43	1.76±0.18	10.87±1.05	34.30±3.21	38.15±3.82	15.07±1.48
Rosmarinic acid	1.91±0.17	3.01±0.33	4.06±0.38	16.10±1.55	5.21±0.45	6.32±0.61	0.52±0.05	13.56±1.23	7.35±0.66	1.19±0.12	3.74±0.26	1.37±0.14
Syringic acid	5.52±0.48	4.84±0.45	5.75±0.61	6.97±0.73	4.63±0.41	3.61±0.34	4.73±0.42	2.91±0.26	5.78±0.54	9.36±0.93	2.36±0.21	5.52±0.51
**Total content**	**111.79±10.67**	**71.57±7.22**	**52.28±5.12**	**110.54±11.24**	**70.41±6.98**	**52.38±5.12**	**45.09±4.11**	**66.71±6.53**	**61.88±6.06**	**62.81±6.24**	**75.05±7.01**	**57.87±5.65**

Table S11. Phenolic acid contents (mg/100 g DW±SD) in extracts from *N. officinale* agar microshoots cultivated under different duration of growth periods on MS media variants with cytokinin – Zea (1 mg/l) and auxins: IAA, IBA, IPA and NAA (1 mg/l). Values represent the mean (±SD) of five experiments (n=5).

**Phenolic acids**	**MS medium variants**											
	**1 mg/l Zea and 1mg/l IAA**			**1 mg/l Zea and 1mg/l IBA**			**1 mg/l Zea and 1mg/l IPA**			**1 mg/l Zea and 1mg/l NAA**		
	**Growth cycle (days)**											
	**10**	**20**	**30**	**10**	**20**	**30**	**10**	**20**	**30**	**10**	**20**	**30**
Caffeic acid	5.45±0.52	5.38±0.49	1.31±0.12	3.10±0.28	4.65±0.52	3.86±0.39	6.62±0.59	6.43±0.56	0.56±0.05	11.43±1.11	5.13±0.43	5.03±0.42
o-Coumaric acid	0.41±0.03	0.27±0.03	0.37±0.04	1.28±0.12	0.63±0.05	0.65±0.07	2.00±0.19	1.74±0.13	0.39±0.04	0.21±0.02	0.26±0.19	1.25±0.11
p-Coumaric acid	6.22±0.59	2.96±0.31	3.57±0.31	4.07±0.38	6.27±0.55	5.89±0.61	14.62±1.34	4.77±0.39	4.25±0.38	5.15±0.49	3.65±0.32	5.54±0.53
Ellagic acid	4.28±0.38	5.02±0.45	3.79±0.35	4.49±0.52	5.43±0.43	3.93±0.41	4.97±0.54	5.78±0.68	4.13±0.42	5.38±0.45	5.21±0.43	5.67±0.53
Ferulic acid	8.25±0.79	11.41±1.17	14.19±1.32	9.11±1.01	11.70±1.23	11.83±1.12	14.96±1.52	12.33±1.16	2.06±0.18	7.90±0.81	9.30±0.88	13.58±1.28
Gallic acid	17.30±1.67	33.78±3.28	24.96±2.39	13.27±1.22	37.90±3.43	10.42±1.09	15.38±1.48	18.27±1.78	15.50±1.64	14.42±1.33	45.01±4.38	25.67±2.21
Isoferulic acid	0.02±0.01	0.06±0.01	0.61±0.05	2.32±0.16	1.87±0.14	1.16±0.11	2.56±0.21	1.05±0.12	0.66±0.07	0.26±0.02	0.18±0.02	3.53±0.32
Protocatechuic acid	21.68±2.09	12.08±1.12	7.23±0.68	27.96±2.83	14.74±1.53	11.32±1.23	18.93±2.01	9.66±0.87	3.22±0.29	61.89±5.97	17.68±1.66	12.04±1.19
Rosmarinic acid	1.66±0.14	2.53±0.24	0.54±0.04	2.48±0.21	2.68±0.33	1.93±0.22	3.53±0.33	4.49±0.44	2.42±0.23	6.88±0.66	1.51±0.21	2.17±0.23
Syringic acid	4.44±0.39	3.57±0.35	4.13±0.35	4.43±0.41	3.79±0.47	4.56±0.42	3.78±0.41	3.39±0.31	5.60±0.62	4.64±0.35	2.77±0.24	5.23±0.48
**Total content**	**69.73±6.67**	**77.04±6.98**	**60.70±5.98**	**72.50±6.92**	**89.67±7.99**	**55.55±5.76**	**87.34±7.84**	**67.91±5.90**	**38.79±4.08**	**118.18±10.43**	**90.70±9.12**	**79.70±6.98**

Table S12. Phenolic acid contents (mg/100 g DW±SD) in extracts from *N. officinale* agitated microshoots cultivated under different duration of growth periods on MS media variants with cytokinins – BA, 2iP, KIN, Zea (1 mg/l) and auxins: IAA, NAA and IBA (1 mg/l). Values represent the mean (±SD) of five experiments (n=5).

Phenolic acids	MS medium variants											
	1 mg/l BA and 1 mg/l NAA		1 mg/l 2iP and 1 mg/l NAA		1 mg/l KIN and 1 mg/l IAA		1 mg/l KIN and 1 mg/l IBA		1 mg/l Zea and 1 mg/l IBA		1 mg/l Zea and 1 mg/l NAA	
	Growth cycle (days)											
	10	20	10	20	10	20	10	20	10	20	10	20
Caffeic acid	0.50±0.05	0.27±0.03	11.57±1.65	1.18±0.12	4.51±0.33	0.02±0.01	8.18±0.77	0.15±0.02	14.37±1.13	8.09±0.77	15.30±1.66	3.48±0.31
o-Coumaric acid	14.91±1.35	3.20±0.28	3.69±0.32	5.87±0.59	1.01±0.11	5.78±0.53	1.23±0.09	11.16±1.21	1.54±0.14	0.82±0.07	2.35±0.19	0.74±0.66
p-Coumaric acid	31.66±2.98	14.61±1.32	17.34±1.86	4.84±0.45	5.91±0.64	5.00±0.48	9.54±0.99	20.20±2.02	10.24±1.11	12.40±1.23	15.93±1.52	12.64±1.26
Ellagic acid	7.16±0.73	3.96±0.41	5.50±0.48	7.88±0.68	3.11±0.28	6.54±0.59	2.93±0.33	8.12±0.79	3.64±0.32	3.94±0.37	3.06±0.28	4.30±0.38
Ferulic acid	38.44±4.12	8.37±0.82	4.99±0.53	13.21±1.28	2.74±0.22	5.41±0.48	2.49±0.21	17.84±1.82	3.91±0.28	1.58±1.64	4.05±0.39	1.97±0.22
Gallic acid	32.69±3.09	29.84±0.28	30.31±3.21	37.41±3.88	31.14±3.54	34.19±3.39	33.78±3.22	37.05±0.44	31.19±3.02	38.47±3.77	53.34±5.12	35.28±3.53
Isoferulic acid	15.17±1.49	2.11±0.18	6.67±0.54	13.37±1.33	1.68±0.14	12.58±1.31	1.11±0.13	10.98±1.21	1.56±0.14	0.02±0.01	2.40±0.19	12.19±1.42
Protocatechuic acid	40.31±4.01	9.37±0.87	23.05±2.56	4.94±0.48	11.90±1.20	8.59±0.82	29.53±3.02	16.23±0.17	22.36±2.10	10.30±1.20	132.26±12.02	17.96±2.01
Rosmarinic acid	4.41±0.38	3.19±0.32	9.41±0.95	36.34±3.71	7.14±0.65	3.24±0.22	3.39±0.34	27.60±3.01	3.66±0.35	2.95±0.33	4.37±0.38	12.74±1.25
Syringic acid	21.50±2.12	7.82±0.69	3.86±0.41	3.39±0.28	1.65±0.18	10.70±1.12	2.32±0.19	11.04±1.11	1.84±0.14	3.01±0.28	3.87±0.41	2.26±0.19
**Total content**	**206.73±19.83**	**82.74±8.12**	**116.38±11.09**	**128.43±13.00**	**70.80±7.12**	**92.06±9.11**	**94.49±9.32**	**160.37±15.92**	**94.31±9.11**	**81.57±8.21**	**236.74±19.30**	**103.55±11.03**

Table S13. The antioxidant activity estimated by CUPRAC, FRAP, DPPH methods and total phenolic F-C method (expressed in mmol TE/100g DW) of *N. officinale* agar *in vitro* cultures cultivated on different MS medium variants and growth cycles. Values represent the mean (±SD) of five experiments (n=5).

MS medium variants	Growth cycles (days)	CUPRAC	FRAP	DPPH	F-C
0 (control)	10	1.60±0.01	1.01±0.07	3.35±0.13	5.00±0.03
	20	2.10±0.08	1.28±0.04	3.17±0.41	6.41±0.58
	30	1.50±0.19	1.04±0.04	3.06±0.29	5.35±0.26
1 mg/l BA and 1 mg/l NAA	10	2.09±0.08	0.90±0.10	3.02±0.50	5.31±0.30
	20	3.26±0.06	0.80±0.10	2.81±0.40	2.70±0.20
	30	2.84±0.11	0.70±0.11	2.70±0.40	2.61±0.30
2 mg/l BA and 1 mg/l NAA	10	2.09±0.08	1.42±0.02	1.41±0.31	5.50±0.04
	20	2.37±0.69	1.03±0.11	0.49±0.07	2.56±0.21
	30	2.26±0.08	0.97±0.02	2.83±0.06	2.42±0.10
1 mg/l BA and 2 mg/l NAA	10	2.22±0.08	1.06±0.11	2.66±035	4.88±0.18
	20	2.34±0.04	1.00±0.06	1.08±0.54	3.55±0.51
	30	2.37±0.07	0.95±0.06	1.24±0.99	3.36±0.31

Table S14. The antioxidant activity estimated by CUPRAC, FRAP, DPPH methods and total phenolic F-C method (expressed in mmol TE/100g DW±SD) of *N. officinale* agar microshoot cultures cultivated on different duration of growth cycles and variants of MS media. Values represent the mean (±SD) of five experiments (n=5).

MS medium variants	Growth cycles (days)	CUPRAC	FRAP	DPPH	F-C
1 mg/l Zea and 1 mg/l NAA	10	3.10±0.10	0.66±0.01	25.24±1.41	8.69±3.44
	20	3.51±0.45	0.74±0.07	20.95±1.49	3.75±0.31
	30	2.92±0.15	0.61±0.04	19.21±1.72	3.23±0.50
1 mg/l Zea and 1 mg/l IBA	10	2.84±0.03	0.63±0.02	24.49±2.87	4.85±0.96
	20	3.98±0.18	0.87±0.05	21.48±0.41	5.64±0.53
	30	3.30±0.34	0.68±0.06	17.91±1.23	3.90±0.81
1 mg/l 2iP and 1 mg/l NAA	10	2.67±0.14	0.52±0.02	20.81±0.71	5.08±0.39
	20	3.49±0.06	0.82±0.01	19.76±1.41	4.64±0.24
	30	2.90±0.18	0.66±0.05	23.57±3.91	4.35±0.70
1 mg/l KIN and 1 mg/l IAA	10	2.98±0.06	0.83±0.06	28.38±0.79	5.54±0.53
	20	3.43±0.06	0.94±0.03	29.69±1.71	6.48±0.21
	30	4.13±0.80	1.03±0.02	30.89±1.82	5.41±0.20
1 mg/l KIN and 1 mg/l IBA	10	2.52±0.10	0.71±0.01	25.65±5.54	3.91±0.17
	20	2.44±0.90	0.83±0.20	18.58±1.25	3.60±0.12
	30	2.72±0.11	0.80±0.02	20.39±3.51	3.74±0.28

Table S15. The antioxidant activity estimated by CUPRAC, FRAP, DPPH methods and total phenolic F-C method (expressed in mmol TE/100g DW±SD) of *N. officinale* agitated microshoot cultures cultivated on different duration of growth cycles and variants of MS media. Values represent the mean (±SD) of five experiments (n=5).

MS medium variants	Growth cycles (days)	CUPRAC	FRAP	DPPH	F-C
1 mg/l BA and 1 mg/l NAA	10	2.65±0.38	0.55±0.02	18.78±1.00	4.19±0.18
	20	2.52±0.15	0.51±0.04	19.57±4.66	4.39±0.22
1 mg/l Zea and 1 mg/l NAA	10	2.77±0.19	0.67±0.03	15.67±1.92	4.01±0.05
	20	4.02±0.03	0.99±0.02	19.73±2.93	3.77±0.12
1 mg/l Zea and 1 mg/l IBA	10	3.91±0.13	0.88±0.01	25.41±2.97	5.10±0.31
	20	3.48±0.87	0.79±0.19	14.93±2.00	3.08±0.83
1 mg/l 2iP and 1 mg/l NAA	10	3.85±0.07	0.85±0.02	20.37±0.30	4.62±0.06
	20	3.38±0.19	0.80±0.05	17.95±1.37	2.49±0.13
1 mg/l KIN and 1 mg/l IAA	10	3.00±0.09	0.60±0.01	14.55±0.96	3.26±0.11
	20	5.26±0.10	1.26±0.04	25.95±4.07	5.24±0.20
1 mg/l KIN and 1 mg/l IBA	10	3.63±0.04	0.78±0.01	19.34±1.81	3.50±0.13
	20	4.77±0.32	1.06±0.05	23.12±2.69	4.44±0.10
